# Rapid, reliable, and reproducible cell fusion assay to quantify SARS-Cov-2 spike interaction with hACE2

**DOI:** 10.1371/journal.ppat.1009683

**Published:** 2021-06-24

**Authors:** Min Zhao, Pei-Yi Su, Danielle A. Castro, Therese N. Tripler, Yingxia Hu, Matthew Cook, Albert I. Ko, Shelli F. Farhadian, Benjamin Israelow, Charles S. Dela Cruz, Yong Xiong, Richard E. Sutton, the Yale IMPACT Research Team

**Affiliations:** 1 Department of Internal Medicine, Section of Infectious Diseases, Yale School of Medicine, New Haven, Connecticut, United States of America; 2 Department of Molecular Biophysics and Biochemistry, Yale School of Medicine, New Haven, Connecticut, United States of America; 3 Department of Epidemiology of Microbial Diseases, Yale School of Public Health, New Haven, Connecticut, United States of America; 4 Department of Internal Medicine, Section of Pulmonary and Critical Care Medicine, Yale School of Medicine, New Haven, Connecticut, United States of America; Washington University School of Medicine, UNITED STATES

## Abstract

COVID-19 is a global crisis of unimagined dimensions. Currently, Remedesivir is only fully licensed FDA therapeutic. A major target of the vaccine effort is the SARS-CoV-2 spike-hACE2 interaction, and assessment of efficacy relies on time consuming neutralization assay. Here, we developed a cell fusion assay based upon spike-hACE2 interaction. The system was tested by transient co-transfection of 293T cells, which demonstrated good correlation with standard spike pseudotyping for inhibition by sera and biologics. Then established stable cell lines were very well behaved and gave even better correlation with pseudotyping results, after a short, overnight co-incubation. Results with the stable cell fusion assay also correlated well with those of a live virus assay. In summary we have established a rapid, reliable, and reproducible cell fusion assay that will serve to complement the other neutralization assays currently in use, is easy to implement in most laboratories, and may serve as the basis for high throughput screens to identify inhibitors of SARS-CoV-2 virus-cell binding and entry.

## Author summary

COVID-19 continues to be a global public health concern. Currently, the in *vitro* assessment of efficacy of any vaccine or therapeutic is based on time-consuming neutralization assays using live or pseudotyped virus at BSL2/BSL3 biocontainment. There are many factors which will affect the neutralization assay, including quality of plasmid, transfection efficiency, virus titer, etc. Here, we developed a novel cell fusion assay based upon spike-hACE2 interaction, which demonstrated excellent correlation with standard spike pseudotyping for inhibition by convalescent sera, cloned antibodies, and biologics, after 16–24 hours co-incubation. It also correlated well with a live virus assay. Other than two stable cell lines, our cell fusion assay requires no specialized research reagents or laboratory equipment and should be easy to adapt for use in most investigative and clinical settings. It will allow for the testing of sera after vaccination or infection, to assess for level of immune protection, and it could be used for high throughput screening for monoclonal antibodies, compounds, and biologics that interfere with virus-cell binding and entry.

## Introduction

A novel severe acute respiratory syndrome coronavirus (SARS-CoV-2) has caused pandemic disease after its emergence at the end of 2019. It was declared as a major global public health issue by WHO in March 2020 [1]. As of May 18^th^ 2021, SARS-CoV-2 has infected nearly 164 million people and has caused close to 3.4 million deaths world-wide (https://coronavirus.jhu.edu/). Currently, many prophylactic and therapeutic strategies are under development to stem this global crisis [2–5], including use of small molecule drugs [6], biologics including interferon [7,8], convalescent sera [9], monoclonal antibodies [10,11], oligonucleotides [12], peptides [13], and vaccines [14,15]. So far, Remdesivir is the only FDA-approved drug for the treatment for COVID-19 patients (https://www.fda.gov/drugs/drug-safety-and-availability/fdas-approval-veklury-remdesivir-treatment-covid-19-science-safety-and-effectiveness), and three vaccines have Emergency Used Authorization (EUA) from the FDA (https://www.fda.gov/emergency-preparedness-and-response/coronavirus-disease-2019-covid-19/covid-19-vaccines), and four vaccines have been formally approved by the EMA (https://www.ema.europa.eu/en/human-regulatory/overview/public-health-threats/coronavirus-disease-covid-19/treatments-vaccines/covid-19-vaccines). However, no other specific drug against the novel coronavirus has been formally approved by the FDA or EMA.

SARS-CoV-2 belongs to the genus *Coronavirus*, in the family *Coronaviridae*. It is an enveloped, non-segmented, positive-sense single-stranded RNA virus [16,17]. Genomic sequences of SARS-CoV-2 and SARS-CoV show high similarity, with amino acid sequence identity being >76% [18]. The genome of SARS-CoV-2 is nearly 30 kb in length, including many open reading frames (ORFs) which express at least 27 proteins [17,19]. Among them, the surface spike glycoprotein (S) plays a key role in viral entry into target cells [20–22]. The receptor-binding subunit S1 attaches to the host cell via the cellular receptor human Angiotensin-Converting Enzyme 2 (hACE2), triggering proteolytic activation of S and subsequent conformational change of the S2 subunit, which facilitates the fusion of viral and cellular membranes [23–25].

An essential element of developing any prophylactic or therapeutic antiviral or vaccine is quantitative measurement of viral replication. The current gold standards for SARS-CoV-2 neutralization include pseudotyping using S and a suitable virus core encoding a reporter [26–29], or inhibition of live virus replication in *vitro* [30,31] or in animal models [32,33]. Pseudotyping requires production of vector supernatants at BSL2 or BSL2+ biocontainment that are then cryostored until use, with assay readout on susceptible cells after a few days; live virus requires BSL3 laboratory and readout by plaque reduction or similar assay 3–5 days after cell or animal infection.

To circumvent some of these issues we developed a cell fusion assay that utilizes S-expressing and hACE2-expressing cells that when mixed together provides a rapid and quantitative readout, based upon human immunodeficiency virus type 1 (HIV) Tat transactivation of an integrated HIV long terminal repeat (LTR)-firefly luciferase gene. Sera from COVID-19+ patients and anti-spike monoclonal antibodies inhibited cell fusion, which correlated highly with pseudotyping and use of live virus results. Soluble receptor binding domain (RBD) of S also inhibited cell fusion to a lesser extent, as did soluble hACE2. This assay is rapid and can be easily modified for high-throughput format, which will facilitate vaccine development, potent monoclonal antibodies screening, and in vitro drug testing against the virus.

## Results

### Development of a quantitative assay for the measurement of S-hACE2-mediated cell fusion in transiently transfected cells

Spike or S protein mediates cell entry into susceptible target cells expressing hACE2 [24,28,34–36]. We first tested whether co-expression of S and hACE2 could result in cell fusion. 293T cells were transiently co-transfected by calcium phosphate co-precipitation with CMV expression plasmids encoding S and hACE2. At 48 h cells were fixed and stained with crystal violet, and there was obvious multinucleate cell formation that was dependent upon both S and hACE2 (S1 Fig). To test whether this occurred when cells were individually transfected, plasmids encoding S and hACE2 were introduced separately into 293Ts along with CMV-Tat and HIV LTR-FFLUC, respectively, and cells mixed at 48 h. The next day cells were fixed, and cell syncytia were observed with higher amounts of plasmid transfected, up to 1 μg (S2A–S2E Fig). In parallel, cells were lysed 24 h after mixing, and we observed a marked increase in RLU (S2F Fig).

Because enumeration of multinucleate cells is at best semi-quantitative, we decided to focus on development of a quantitative cell fusion assay, based upon HIV LTR activation by HIV Tat. As an initial experiment 293T cells were transiently co-transfected with a plasmid encoding either empty vector (EV), S driven by CMV promoter with or without protease TMPRSS2, or VSV-G along with HIV long terminal repeat driving firefly luciferase (HIV LTR-FFLUC). After 48 hours, these cells were incubated with target 293T cells that had been co-transfected with a plasmid encoding EV or hACE2 with or without TMPRSS2, along with HIV Tat, each driven by the CMV promoter. After another 48 h cells were lysed and FFLUC activity quantified by luminometry. We observed a ~100-fold increase in RLU when both S and hACE2 were each separately transfected, consistent with cell fusion (S3 Fig). As expected, when VSV-G was introduced the increase in RLU occurred independent of expression of hACE2. Interestingly, co-transfection of TRMPRSS2, the protease thought to activate S for cell fusion [34,37], did not further increase RLU activity, when introduced along with either S or hACE2 in the producers or targets, respectively (S3 Fig). This may be because 293Ts already express this protease or presence of this specific protease is not required for cell fusion in these cells.

### Studies with soluble hACE2 and Spike ectodomain

**Fig 1:**
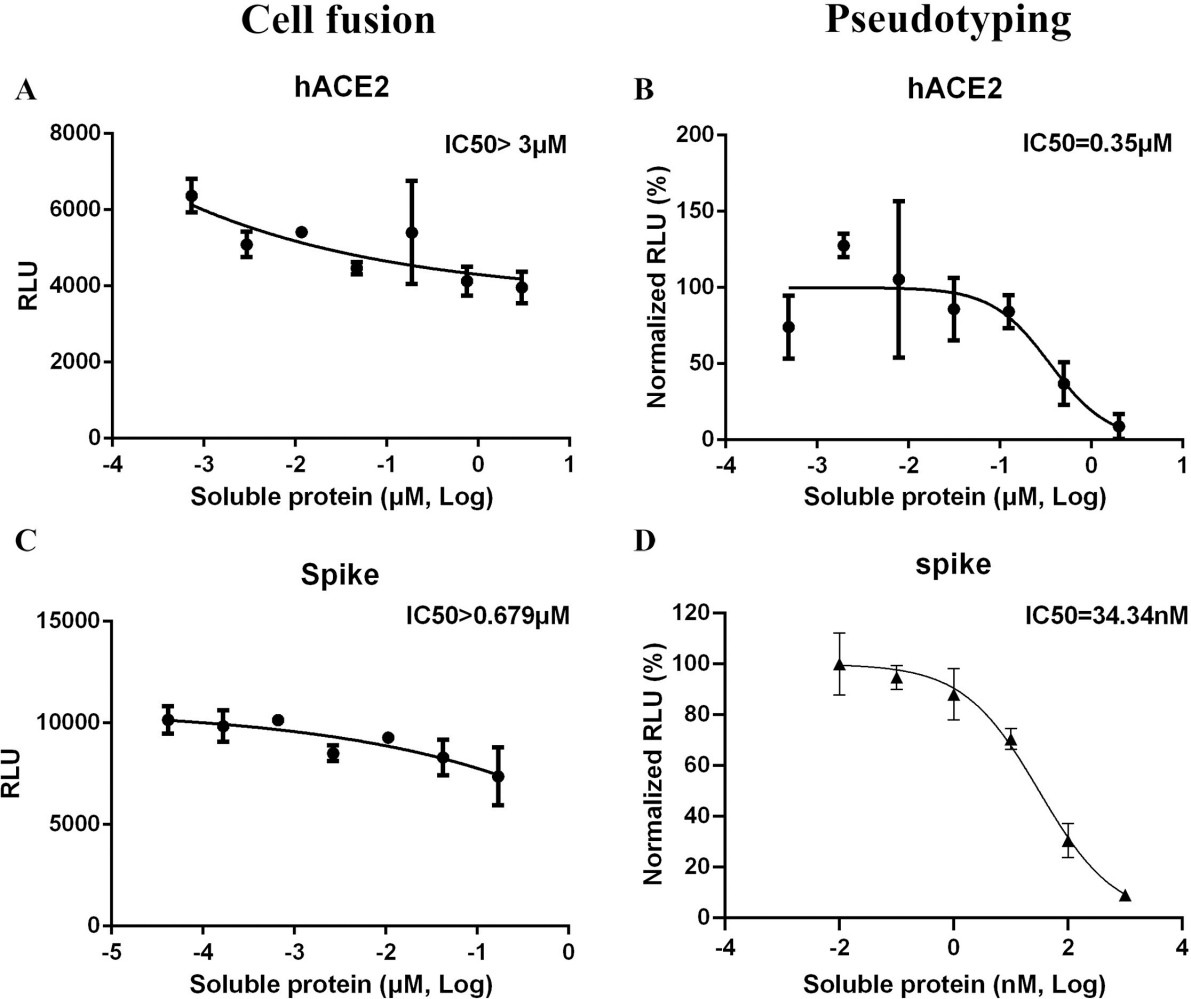
Soluble proteins inhibit cell fusion in the transient system. Increasing concentrations of soluble hACE2 (A) and soluble Spike RBD (C) were pre-incubated with spike-expressing cells or hACE2-expressing target cells as in S3 Fig, and RLU quantified the next day. Spike pseudotyping in the presence of soluble hACE2 (B) and soluble Spike RBD (D) was performed using 293T-hACE2 targets, with RLU measured at ~72 h.

We wished to test the validity of the cell fusion assay by using putative biological inhibitors. To characterize the cell fusion assay, increasing concentrations of soluble hACE2 protein were pre-incubated with S-expressing cells for 1 h prior to mixing with hACE2-expressing cells. Soluble hACE2 was able to inhibit cell fusion at a relatively high concentration, with calculated IC_50_ of >3μM (Fig 1A). Entry of pseudotyped particles was also inhibited by soluble hACE2, with an IC_50_ of 350 nM (Fig 1B).

We performed similar experiments with purified ectodomain of S protein (Fig 1C and 1D). Soluble ectodomain of S was pre-incubated with S-expressing cells prior to mixing with hACE2 expressing cells, with cell fusion measured the next day. Calculated IC_50_ for soluble ectodomain of S was >679 nM (Fig 1C), whereas for pseudotyped particles IC_50_ was ~34 nM (Fig 1D). This suggests that, compared to pseudotyping, cell fusion is more difficult to inhibit by at least 10- to 20-fold, using either of these biomolecules.

### Serum from COVID-19+ patients inhibit cell fusion and syncytia formation

**Fig 2:**
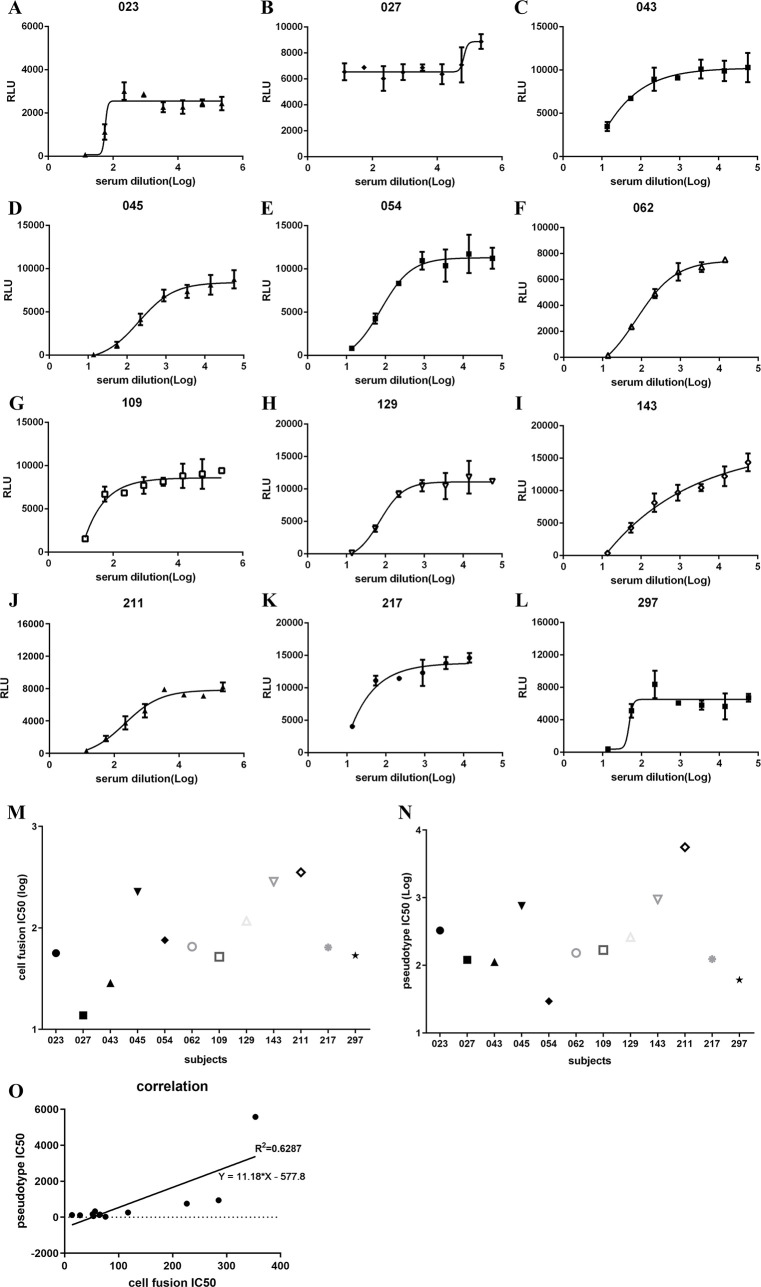
COVID-19+ convalescent and acute illness sera inhibit cell fusion in the transient system. Four-fold serially-diluted serum samples were pre-incubated with spike-expressing producer cells for 1 hour, then hACE2-expressing target cells were added, as per S3 Fig. RLU was measured the next day **(**A-L), and IC_50_ values calculated for each (M). Similarly, IC_50_ values were calculated for pseudotyping, performed as in Fig 1 (N). Correlation between IC_50_ values for the transient cell fusion and S-pseudotyping (O).

A convenience sampling of convalescent and acute illness sera from 12 COVID-19+ subjects, some with acute infection and some during recovery phase, were also tested in the cell fusion assay. Demographic and clinical characteristics of the subjects are shown in S1 Table. With a single exception, all tested sera inhibited cell fusion at varying titers (Fig 2A–2L). Setting aside outlier 027, IC_50_ titers for the cell fusion assay varied between 13.75 and 353.5 (Fig 2M). Serum was also tested in the pseudotyping assay (Fig 2N), and there was a high degree of correlation between cell fusion and pseudotyping IC_50_ titers (Fig 2O). Similar to what we had observed with soluble hACE2, inhibition of cell fusion required more sera than inhibition of pseudotyping. This suggests that, depending on the biologic or drug being tested, compared to pseudotyping the cell fusion assay may be a more rigorous test of viral inhibition.

### Development of stable cell lines to quantify S-hACE2 cell fusion

The data presented above demonstrate quantitation of cell fusion in transiently transfected cells. Because transient transfection is relatively complicated, occasionally unreliable, and not amenable to high throughput use, we decided to develop stable cell lines that could quantify cell fusion rapidly, reliably, and reproducibly. First, cell lines stably expressing S (TZMbl-Spike) or hACE2 (HOS-3734 or 3742) were generated, as described in **Materials and Methods**. TZMbls, based upon HeLa cells, have integrated HIV LTR-FFLUC and HIV LTR-LacZ cassettes that are both Tat-responsive; they have been widely used in the HIV field for titering virus stocks and performing pseudotyping assays, especially to measure neutralization by sera and cloned antibodies. The HOS cells used here had been transduced with a third-generation HIV vector in which *tat* remains intact. Importantly, unlike 293Ts, HOS cells do not naturally fuse with TZMbl cells. Expression of the expected 160 kD hACE2 protein in both HOS-3734 and HOS-3742 cells was verified by immunoblotting (S4A Fig). Expression of S in TZMbl-S cells was observed by using anti-FLAG antibody on immunoblots (S4B Fig). Syncytia formation was observed 24 h post incubation when these two cell types were mixed together (S5A–S5F Fig). The syncytia were LacZ+, as expected (S6 Fig). Similarly, the extent of cell fusion could be quantified by measuring RLU (S5G Fig). Only background levels of RLU were observed when TZMbls without S were used. Markedly increased RLU were measured when the hACE2 expressing cells (either HOS-3734 or HOS-3742) were co-cultured with TZMbl-S cells. Interestingly, there was detectable RLU above background when TZMbl-S cells were mixed with HOS cells carrying empty vector, indicating that either i) HOS cells express another protein that can function as a receptor, ii) HOS cells express a very low, undetectable level of hACE2, or iii) limited cell fusion can occur in the presence of S, independent of the expression of a specific cellular receptor. A time course experiment demonstrated that RLU clearly increased by 20 h and peaked at around 30 h after co-culture (S5H Fig).

### Further characterization of the stable cell line cell fusion system

**Fig 3:**
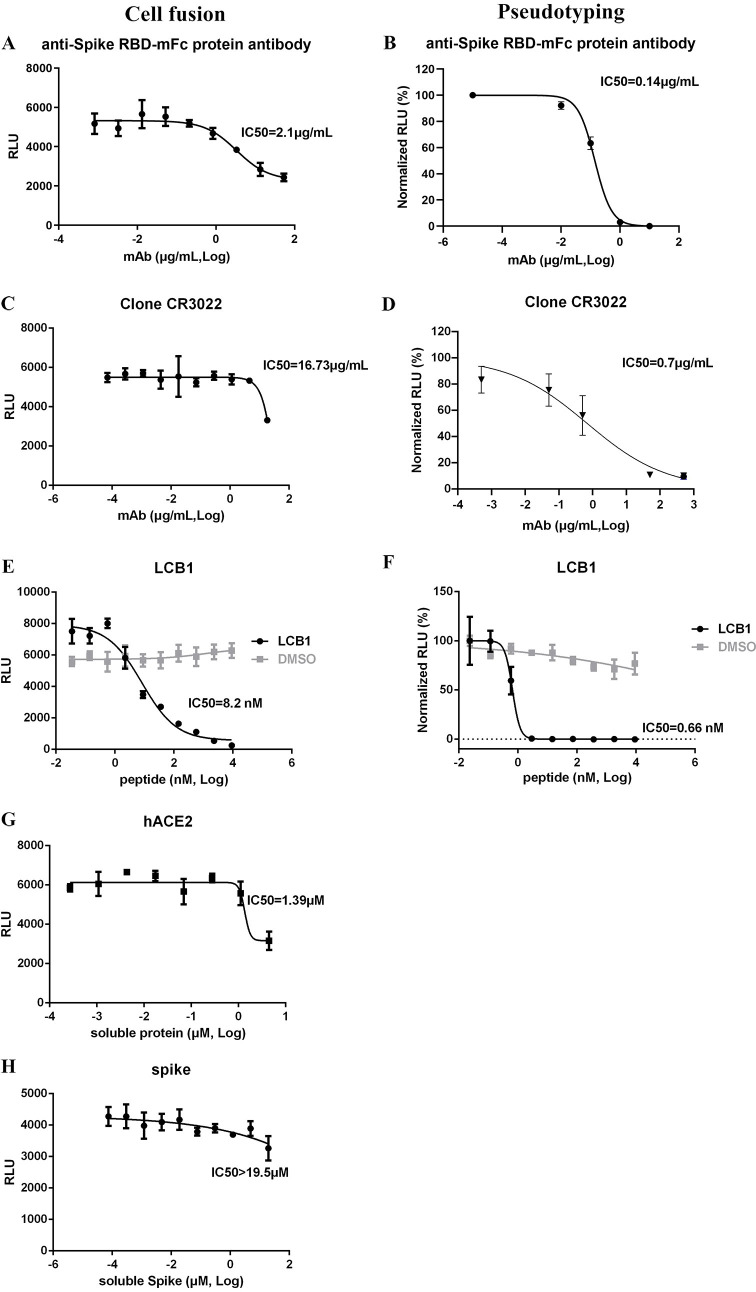
Monoclonal antibodies, peptide LCB1, and soluble proteins inhibit cell fusion in the stable system. Serially diluted anti-Spike RBD-mFc protein antibody(A), Clone CR3022 (C), peptide LCB1 (E), soluble hACE2 (G) and soluble Spike protein (H) were incubated with producer cells (TZMbl-Spike) for 1 h. Target cells (HOS-3734) were then added, with RLU measured the next day. Serially diluted Spike RBD-mFc antibody (B), Clone CR3022 (D) or peptide LCB1 (F) were incubated with S-pseudotyped viral particles, and then added to target 293T-hACE2 cells, with RLU measured at ~72 h. IC_50_ values are shown.

Anti-spike monoclonal antibodies, soluble hACE2 and spike RBD, and convalescent/acute illness human sera were tested in the stable cell line cell fusion system. Both commercial monoclonal antibody against Spike RBD mFc protein (Fig 3A) and clone CR3022 (Fig 3C) were able to inhibit cell fusion in the stable system, and the IC_50_ values were 2.1 and 16.73 μg/mL, respectively, whereas in the pseudotyping assay IC_50_ values were 15–20 fold less at 0.14 and 0.70 μg/mL, respectively (Fig 3B and 3D). Peptide LCB1 was reported to efficiently neutralize pseudotyped Sars-Cov-2 virus entry [38]. When we tested its ability to inhibit cell fusion the IC_50_ was 8.2 nM (Fig 3E), which is 12-fold greater than the IC_50_ value for inhibiting pseudotyping (0.66 nM; Fig 3F). Furthermore, a time of addition experiment demonstrated that LCB1 could efficiently inhibit cell fusion at -1 and 0 h before target-producer cell co-incubation (S7 Fig). Soluble hACE2 inhibited cell fusion at higher concentrations, with an IC_50_ of 1.39 μM (Fig 3G), whereas soluble spike RBD was virtually inactive (IC_50_ >19.5 μM, Fig 3H).

**Fig 4:**
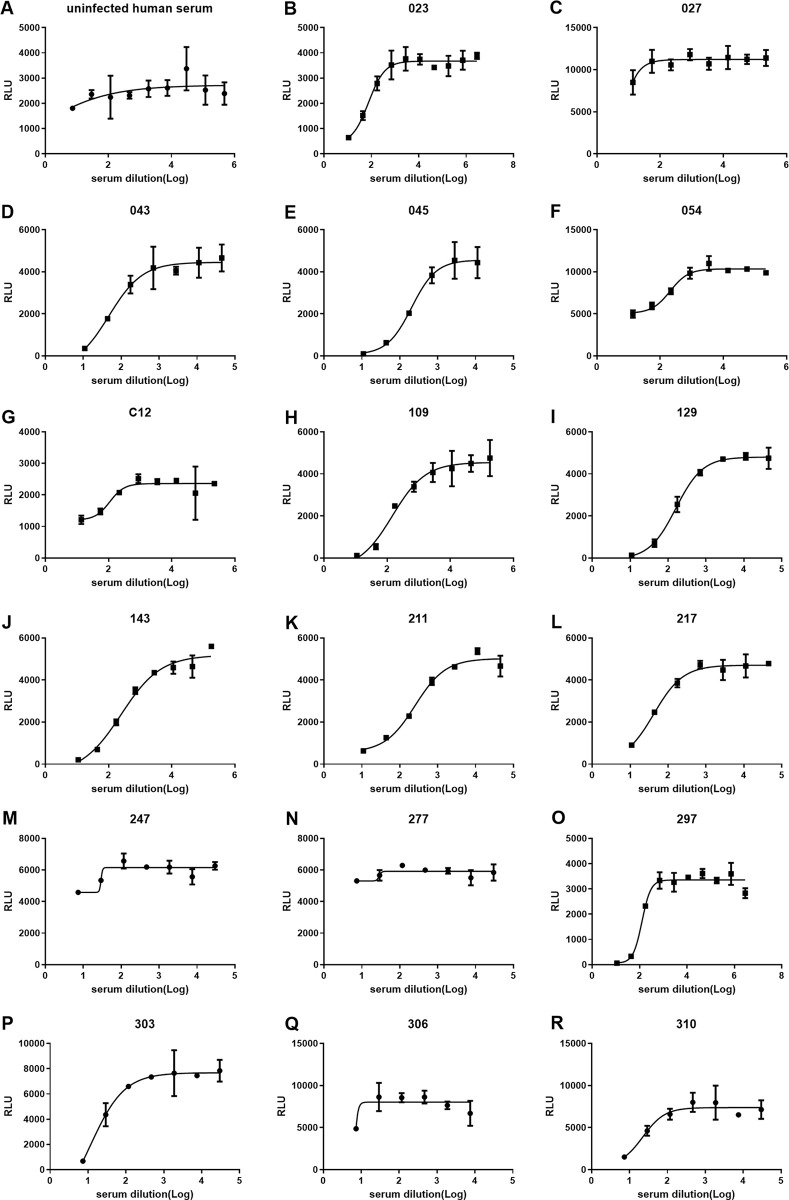
COVID-19+ convalescent and acute illness sera inhibit cell fusion in the stable system. Four-fold serially-diluted sera were pre-incubated with TZMbl-Spike producer cells for 1 h, then HOS-3734 target cells were added. RLU was measured the next day. (A) uninfected human serum. (B-R) convalescent and acute phase sera from COVID 19 (+) patients.

**Fig 5:**
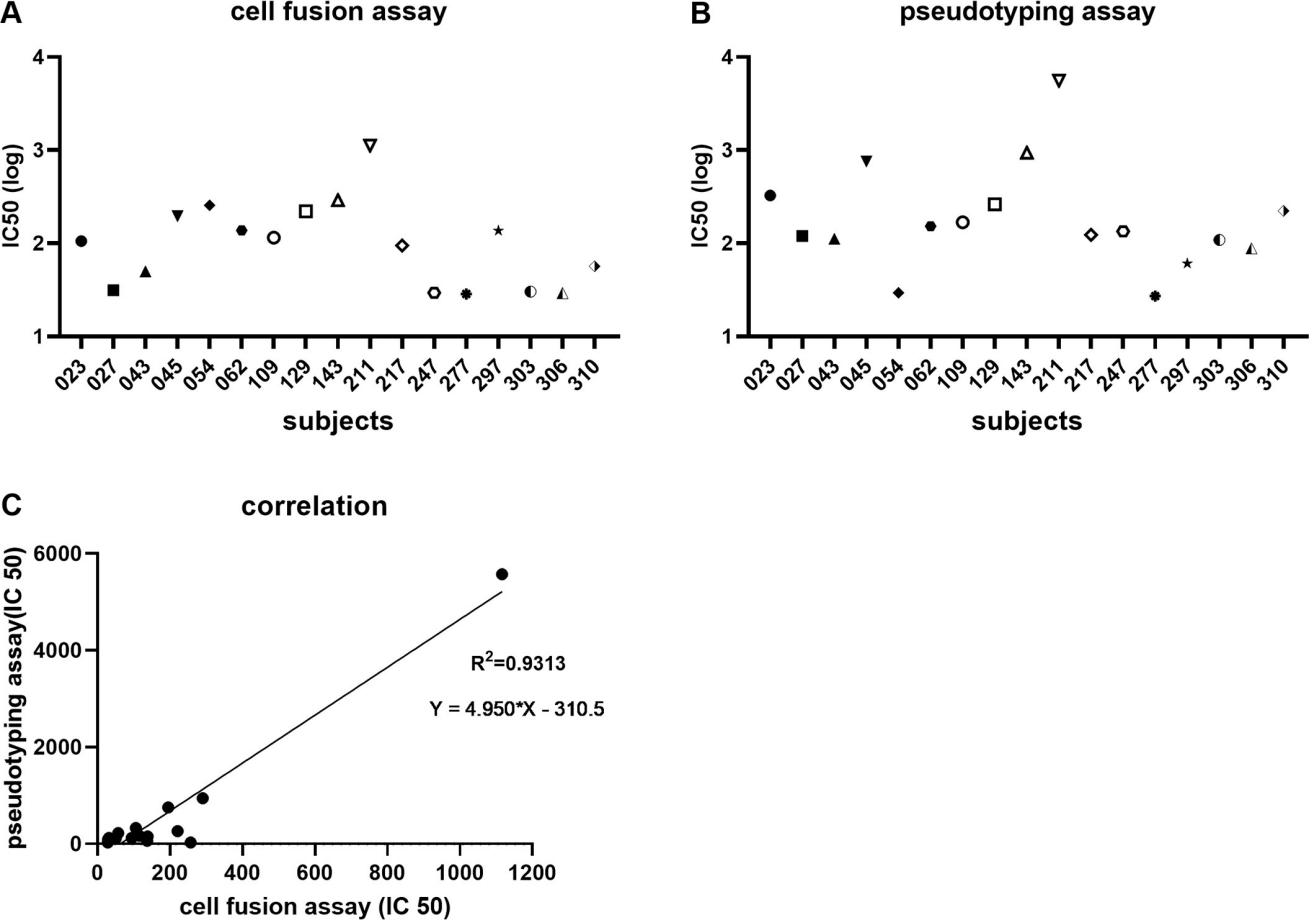
Correlation between the cell fusion and pseudotyping assays. IC_50_ values of the 17 serum samples to inhibit cell-cell fusion between TZMbl-Spike producer and HOS-3734 target cells are shown; note log scale of Y-axis (A). IC_50_ values of the 17 serum samples to inhibit pseudotying virus entry are shown (B). Correlation between cell fusion and pseudotyping IC_50_ values (C).

We randomly picked another 5 acute and convalescent sera such that 17 were tested using stable cell line system (HOS-3734). Clinical and demographic characteristics of these subjects are included in S1 Table. Again, 13 out of the 17 convalescent sera were able to inhibit cell fusion, with the exception of subjects 027, 247, 277, and 306 (Fig 4); IC_50_ values are shown in Fig 5A. And IC_50_ values for pseudotyping assay are shown in Fig 5B. For these samples there was an extremely robust correlation between IC_50_ values of the stable cell fusion and pseudotyping assays (R^2^ = 0.93; Fig 5C). Again, for all the tested sera the cell fusion IC_50_ titers were lower than those of pseudotyping. Additionally 12 sera were tested on the HOS-3742 stable cell line system (S8 Fig), and results were consistent with those using HOS-3734 cells.

A total of 7 sera were selected at random to test neutralization activity using replication-competent SARS-CoV-2-nLuc-GFP reporter virus (S9 Fig) on VeroE6 cells, and IC_50_ values were calculated. Based on those results IC_50_ values were correlated between the stable cell fusion and pseudotying assays (R^2^ = 0.997; S10A Fig), between pseudotyping and replication-competent virus assays (R^2^ = 0.82; S10B Fig), and between cell fusion and replication-competent virus assays (R^2^ = 0.79; S10C Fig).

**Fig 6:**
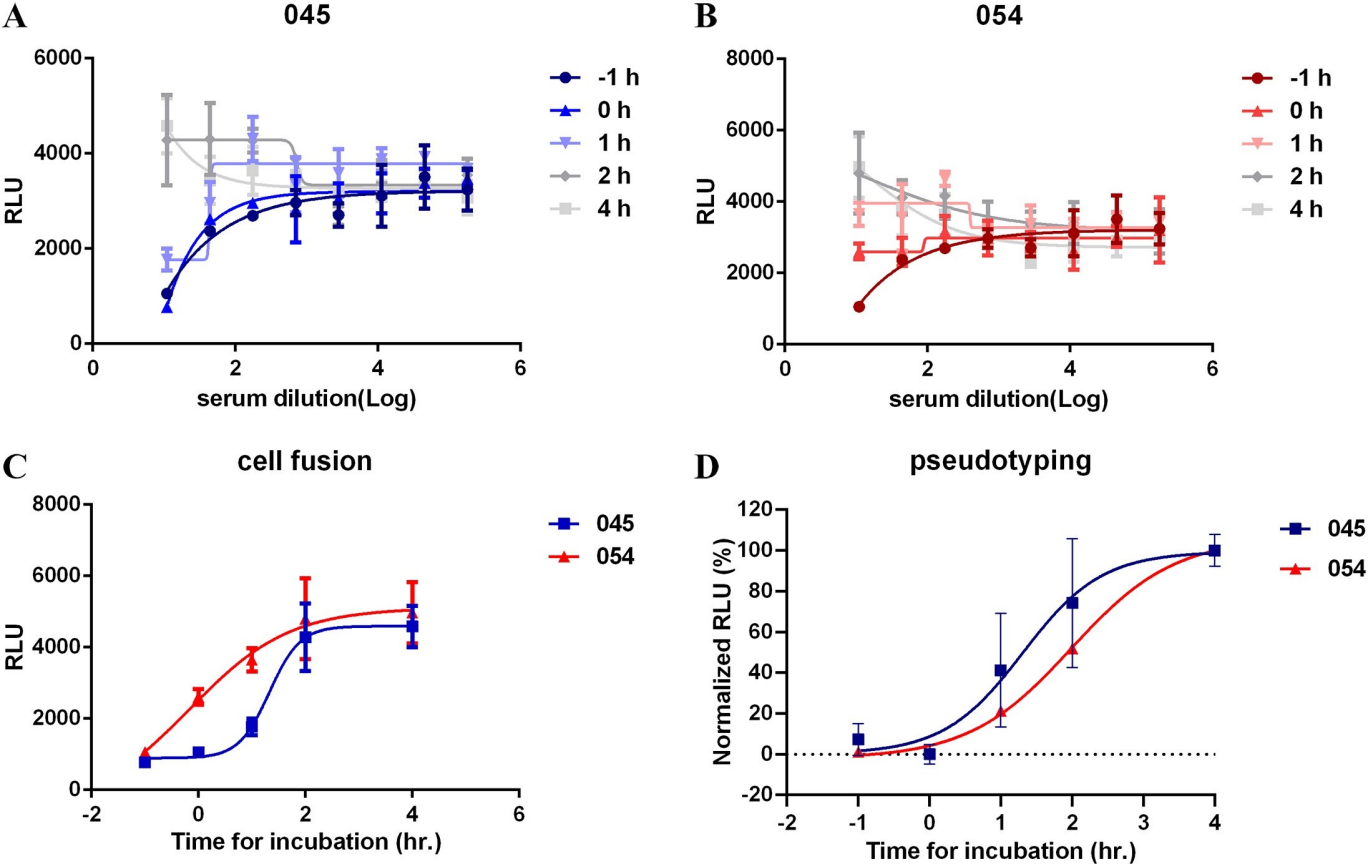
Addition of sera after co-culture minimally inhibits cell fusion. Four-fold serially-diluted sera 045 (A) or 054 (B) was added 1 h before or at 0, 1, 2, or 4 h after co-culture of HOS-3734 and TZMbl-S cells. RLU was measured the next day. (C) Time course assay of inhibiting cell fusion at highest concentration of sera. (D) Times course assay of inhibiting S-pseudotyped viral particles entry, also using a high concentration of sera.

Furthermore, a time of addition experiment demonstrated that convalescent sera 045 could efficiently inhibit cell fusion at -1 and 0 h before target-producer cell co-incubation (Fig 6A and 6C), and convalescent sera 054 could efficiently inhibit cell fusion at -1 h (Fig 6B and 6C). Beyond 2 h of co-incubation inhibition of cell fusion by sera 045 or 054 was minimal to non-existent (Fig 6C). As shown in Fig 6D, data from the cell fusion assay is largely consistent with that of pseudotyping, although it appears that even at time of addition = 2 h of both sera there is some degree of inhibition of pseudotyping.

In order to test the reproducibility of the cell fusion assay, LCB1 peptide and murine mAb against Spike protein (details of this mAb to be published separately) were each tested 5 times in 5 independent experiments performed at separate, discrete times over a 6 month period, with respective, aggregate IC_50_s of 0.096±0.04 μg/mL and 5.52±1.64 nM (see S11 Fig for data for LCB1 peptide). Thus, the data suggest that the stable cell lines are reliably well-behaved and may be a suitable platform for high-throughput screening of sera, monoclonal antibodies,biologics, and small molecules that inhibit SARS-CoV-2 virus entry into cells.

## Discussion

Current prophylactic and therapeutic efforts to stem the global COVID-19 pandemic include vaccines, biologics, and small molecules [2,15,39–42]. Virtually all of the vaccines target SARS-CoV-2 spike-hACE2 interaction, as do many of the other agents, in order to prevent virus binding and entry into target, receptor-bearing cells [15,43,44]. Current ‘gold standards’ to quantify the efficacy of such measures include inhibition of target cell infection by pseudotyped particles and fully replication-competent virus [6,28]. Both of these methods require consistent and reliable production and cryostorage of viral particles, use under BSL2+ or BSL3 biocontainment, and readout is typically performed several days later (although there are exceptions; see [45]). Herein we borrowed a page from the HIV playbook and report the development of stable cell lines in which quantification of cell fusion is available overnight after co-incubating the cells.

We were impressed with the degree of syncytia formation after co-expression of S and hACE2 (S1 Fig), which also occurred when the protein products were separately expressed and the cells subsequently mixed (S2 Fig). Because counting multinucleate giant cells is at best semi-quantitative, we next used a reporter assay after transient transfection that relies upon HIV Tat trans-activating the HIV LTR [46]. Although transient transfection did give somewhat reliable and rapid results which correlated with S pseudotyping using HIV cores (Figs 1 and 2), it required repeated, fresh cell transfections of multiple plasmids, which is inconvenient, variable, and may not be reproducible if widely employed. The stable cell lines that we have developed, on the other hand, are facile to use and very reliable. They behave very consistently over many months, even in the absence of antibiotic selection. The actual RLU values obtained are quite high for cells plated in 96-well format and the dynamic range coupled with low standard deviations, based upon inhibition by sera and monoclonal antibodies tested, should be sufficient for a high throughput screen to identify inhibitors of S and hACE2 interaction.

At present it is unknown precisely how SARS-CoV-2 spreads and replicates in human tissues, including the respiratory tract [47]. Whether cell-free versus cell-to-cell transmission of this virus occurs in vivo is an open question; generally speaking cell-to-cell transmission of virus via a virological synapse is much more efficient than infection by cell free virus [48–53]. It is conceivable that SARS-CoV-2 causes cell fusion between S and hACE2-expressing cells in man [28,54]. There is increasing histopathological evidence of giant, multinucleate respiratory epithelial and intra-alveolar cells in COVID-19+ patients [55–57]. Although some of those cells had features suggestive of being virally-infected, definitive evidence is lacking. It should be made clear that our results here are not meant to address or answer that interesting question, but rather to demonstrate that a quantitative assay based upon cell fusion has been established for spike-hACE2 interaction.

That the cell fusion and pseudotyping IC_50_ values for the convalescent and acute illness COVID-19+ sera (Figs 2 and 4 and 5 and S10) correlated highly suggests that the cell fusion assay is indeed measuring the ability of S and hACE2 to interact. Unsurprisingly, there was also significant correlation between the cell fusion, pseudotyping, and live virus assays in terms of IC_50_titers, although certainly with additional serum testing discrepancies may arise which might be worth scientific pursuit. It is also not surprising that more sera and antibody is needed to inhibit the cells from fusing. Although we have not attempted to quantify the numbers of spike and hACE2 proteins on the stable cell lines, typical expression levels would be >10,000 molecules of each, whereas an intact SARS-CoV-2 virion may have just a few dozen spike trimers [58–60], depending upon its source and how it is made. Nor is it known how many S-hACE2 interactions are required to trigger irreversible pore formation and subsequent cell-cell or cell-virus fusion. Although we do not know the relative cell surface density of either S or receptor on either stable cell line, based upon stoichiometry alone it is quite likely that inhibition of cell-cell fusion is much more difficult than inhibition of virus-cell fusion, whether the latter is due to pseudotyped or replication-competent virus. Here, this is borne out in the much higher amounts of sera and antibody required to achieve 50% inhibition of fusion (Figs 2–5). More importantly, however, the IC_50_ values for both assays are highly correlated (Figs 2 and 5 and S10), suggesting that the cell fusion assay has utility and in fact may be a more rigorous test for the inhibitory power of any antibody, serum, biologic, or even small molecule, when compared to other cell-based assays that rely on production of virus.

In addition to sera and monoclonal antibodies, we tested two other biologics—purified, soluble hACE2 and RBD of spike (Figs 1 and 3). Soluble hACE2, stabilized by an Fc domain for a longer half-life in plasma, has been proposed as a potential therapeutic agent, especially since the S-hACE2 interaction has a K_D_ in the low nanomolar range [61,62]. hACE2 has now been subjected to saturation mutagenesis of the RBD-interacting residues, and several hACE2 variants have been identified that have even greater affinity for RBD (K_D_ in the picomolar range) [63]. Here, we tested wt, soluble hACE2 (Figs 1A and 3G), and although it inhibited cell fusion the amounts required to do so were relatively high. Whether soluble or stabilized hACE2 variants are more potent against cell fusion will require further testing. At the moment soluble RBD and variants thereof have only been proposed and tested as potential vaccine candidates [64], not as therapeutics, so RBD testing here was purely academic. As anticipated, RBD did interfere with both pseudotyping and cell fusion, although for the latter the degree of inhibition was marginal at best. The fact that both soluble hACE2 and RBD inhibited cell fusion further corroborates the validity of the assay. A third biologic, peptide LCB1, also had low nM IC_50_ inhibitory activity in the cell fusion assay, as would be predicted based upon pseudotyping results.

The cell fusion assay was highly reproducible in that repeated testing of both an inhibitory peptide and a murine mAb gave extremely consistent results over a period of many months, with at most a 2-fold variance in IC_50_ values. This suggests that the cell fusion assay, when performed by other investigators throughout the world, should also behave similarly, since all that needs to be done is co-culture of two stable cell lines, without the need for making, storing, or testing any virus, live or pseudotyped. This will allow straightforward comparisons of the potency of various virus entry inhibitors between disparate laboratories.

In summary, we have developed a novel SARS-CoV-2 spike-hACE2 cell fusion assay that is rapid, reliable, and reproducible. Other than two stable, well-behaved cell lines it requires no specialized research reagents or laboratory equipment and should be easy to adapt for use in most investigative and clinical settings. It will allow for the testing of sera after vaccination or infection, to assess for level of immune protection, and it could be used for high throughput screening for compounds and biologics that interfere with virus-cell binding and entry.

## Material and methods

### Ethics statement

COVID-19+ convalescent serum was obtained from YNHH hospital (IMPACT research team). IMPACT study used the Yale university institutional review board (IRB) or Biomedical (HIC). And the approval was granted by the Yale HIC. All informed, written consent was obtained from all subjects.

### Vectors and plasmids

CMV-driven expression plasmid for S that was also FLAG-tagged at the COOH terminus (pcDNA-SARS-CoV-2-S) was a kind gift of Craig Wilen (Yale). Plasmid encoding human ACE2 (hACE2) was obtained from Addgene (hACE2; catalog #1786). The hACE2 2.6 kbp ORF was blunt-subcloned into pShuttle (Clontech) to make pShuttle-hACE2. It was also blunt-cloned into a third generation HIV vector 3’ of CMV promoter and 5’ of an IRES-puro^r^ cassette to generate pHIV-CMV-hACE2-IRES-Puro. A separate third generation HIV vector pLV-EF1a-hACE2-cMYC-FLAG-IRES-Puro was obtained from Craig Wilen. pSV-Tat, pCMV-Tat, and pLTR-LUC were kind gifts of Andrew Rice (Baylor College of Medicine). Spike from pcDNA-SARS-CoV-2-S was inserted into a *piggybac* transposon (originally obtained from Matt Wilson of Baylor, along with the transposase plasmid pCMV-*piggybac*) that had been modified to encode a CMV-IRES-*bsd*^r^ cassette; resultant plasmid was named pT-PB-SARS-CoV-2-Spike-IRES-Blasti.

### Cell lines

Human embryonic kidney cell line 293T (#CRL-3216), human bone cell line HOS (#CRL-1543) were originally purchased from ATCC. Africa green monkey kidney cell line VeroE6 (#CRL-1586) were purchased from ATCC. TZMbl cells (#JC53BL-13) were obtained from the NIH AIDS Reagent Program. The HOS cells were stably transduced with a third generation HIV vector encoding *tat*, along with eGFP, mRFP, and bleomycin resistance gene; they were maintained in 200–400 μg/mL phleomycin (Invivogen) and were eGFP and mRFP-positive by flow cytometry. hACE2 was subsequently introduced by VSV G-mediated HIV-based transduction using pLV-EF1a-hACE2-cMYC-FLAG-IRES-Puro and pHIV-CMV-hACE2-IRES-Puro, respectively, to produce HOS-3734 and HOS-3742 cells, both cell lines maintained in selection using 10 μg/mL puromycin (Sigma-Aldrich). Those two vectors were also introduced into 293T cells to produce 293T-hACE2 cells for use with pseudotyped particles. Control HOS-2072 cells were created by transducing them with the empty vector HIV-CMV-IRES-puro and maintaining them in 10 μg/mL puromycin. TZMbl cells stably expressing S were created by co-transfecting TZMbl cells with pT-PB-SARS-CoV-2-Spike-IRES-Blasti along with pCMV-*piggybac* and resistant cells selected with 10 μg/mL blasticidin (Invivogen). The control stable cell line not expressing S was generated by co-transfecting pCMV-*piggybac* with pT-pB-IRES-Blasti and selecting for blasticidin-resistant cells.

### Serum, antibodies, soluble RBD, and soluble hACE2

COVID-19+ convalescent and acute phase sera were obtained from YNHH (IMPACT research team). Human uninfected serum was purchased from Sigma-Aldrich. Goat anti-hACE2 polyclonal antisera (#AF933) was purchased from R&D Systems and was used at 1:2000. Rabbit anti-goat antisera was purchased from Sigma-Aldrich and used at 1:20000. Mouse-anti-FLAG monoclonal antibody was purchased from Sigma-Aldrich, as was goat anti-mouse IgG. Monoclonal human IgG1 antibody against SARS-CoV and SARS-CoV-2 Spike (clone CR3022) were purchased from InvivoGen. Murine anti-SARS-CoV-2 Spike antibody (SARS-CoV-2 Spike RBD-mFc protein) was purchased from Sino Biological (# 40592-MM57). 56-mer peptide LCB1 (DKEWILQKIYEIMRLLDELGHAEASMRVSDLIYEFMKKGDERLLEEAERLLEEVER) was synthesized by ABI Scientific and dissolved in DMSO and stored at -80°C prior to use.

Spike RBD domain plasmid was a gift of David Veesler (University of Washington) and the human ACE2 ectodomain plasmid was a gift of Jason McLellan (University of Texas at Austin). Both constructs have histidine tags to allow Ni column purification. Plasmids were transfected into Expi293F cells (ThermoFisher) using the manufacturer’s protocol. Culture supernatant containing the secreted protein was harvested after 3–4 days, and dialyzed against its Ni-NTA binding buffer. Protein was then purified through Ni-NTA affinity chromatography (Qiagen), and size exclusion chromatography (SEC) using a Superdex 200 column (GE Life Sciences) equilibrated with its SEC buffer (listed below). SDS-PAGE was used to monitor purification steps and ensure protein homogeneity. Peak fractions were concentrated, flash frozen and stored at -80°C for future use.

### Cell fusion assay

For the transient transfection cell fusion assay producer 293T cells were co-transfected with pSV-TAT or pCMV-Tat and pcDNA-SARS-CoV-2-S, while target 293T cells were co-transfected with pLTR-LUC and pShuttle-hACE2 or hACE2 plasmids. At 48 h transfected cells were lifted, mixed 1:1, and after another 16–24 h cells were lysed and RLU measured by plate reader in 96-well format as described. Images of cell syncytia were captured with a Nikon TE2000 epifluorescence microscope running MetaMorph software. For the stable cell fusion assay HOS cell lines stably expressing HIV Tat and hACE2 (termed HOS-3734 and HOS-3742) were mixed 1:1 with TZMbl cells stably expressing S. After 16–24 h FFLUC activity was measured and syncytia images captured as described above. To observe LacZ activity, after cell fixation X-gal substrate was used as described. All experiments were performed with biological duplicates and repeated at least twice.

### Cell fusion inhibition by serum or antibodies

Producer and target cells were generated as described above. With regards to the transient system, producer cells were lifted 48 h post transfection and 10^4^ cells were resuspended in 100 μL medium per well in 96-well plates. Serial dilutions of antibody or serum were then added to producer cells and incubated at 37°C for 1 hour. At that point 10^4^ target cells (50 μL per well) that had been transfected 48 h prior were added to producer cells, and after another 24 h cells were lysed in 0.1 mL and RLU measured. With regards to the stable cell lines, 10^4^ producer cells (TZMbl-Spike) in 100 μL of medium in the absence of blasticidin were seeded in 96 well plates. After 24 h, 70 μL of four-fold serially diluted antibody or serum was added into producer cells and incubated at 37°C for 1 hour. Serum and antibody concentrations were the same as above. At that time 10^4^ target cells (HOS-3734 or HOS-3742) in 50 μL medium were then added to the producer cells, and after another 24 h cells were lysed in 0.1 mL and RLU measured. Data were analyzed with non-linear regression using GraphPad Prism to determine the neutralization curve and the IC_50_ values calculated.

### Inhibition of cell fusion with soluble hACE2, Spike RBD and peptide LCB1

Seventy μL of four-fold serially diluted, purified soluble hACE2, spike RBD or peptide LCB1 were added to 96-well plates, which were seeded with 10^4^ producer or target cells in 100 μL per well, respectively. hACE2 dilutions began at 10 μM, spike RBD at 43 μM, and peptide LCB1 at 9.176 μM. After 1 h 0.5×10^4^ target or producer cells in 50 μL were added per well. After 16–24 h 100 μL of lysis buffer was added to each well and RLU measured. The assay was performed for both transient and stable cell lines.

### Time course experiment

96 well plates were seeded with 10^4^ HOS-3734 cells in 100 μL per well. Four-fold serially diluted serum (045 or 054) or LCB1 were added at -1, 0, +1, +2, and +4 h relative to TZMbl-S cells addition. After 16–24 h cells were lysed and RLU measured.

### Pseudotyped virus neutralization assay

Pseudotyped HIV-FFLUC was produced as previously described [65] but using pcDNA-SARS-CoV-2-S instead of VSV G or HIV Env plasmid. If necessary, pseudotyped particles were concentrated by ultracentrifugation. Serum from clinical samples, antibodies, or soluble proteins (hACE2 or Spike RBD) were serially diluted as indicated and pre-incubated with SARS-CoV-2 pseudotyped particles for 1 h at 37°C before inoculation onto 293T-hACE2 target cells. After an overnight incubation fresh medium was added. After another 48 h cells were lysed and RLU measured. IC_50_ values of sera, antibodies or soluble proteins were calculated using GraphPad Prism software. Non-linear regression with normalized response model was applied.

### Replication-competent SARS-CoV-2 Nano luciferase neutralization assay

Experiments using infectious SARS-CoV-2 were performed in a Biosafety Level 3 facility, licensed by the State of Connecticut and Yale University. Nano luciferase expressing SARS-CoV-2 infectious clone (“icSARS-CoV-2-nLuc-GFP”) was previously described and generously provided by Ralph Baric (UNC) [30]. P3 viral stock was generated in VeroE6 cells (cultured in DMEM containing 5%FBS, 1% sodium pyruvate, and 1% Penicillin-Streptomycin) by infecting at a MOI 0.01 for two-three days to generate a working stock. After incubation the supernatant was clarified by centrifugation (500g × 5min) and filtered through a 0.45-micron filter, and virus titer was determined by plaque assay on VeroE6 cells as previously described [66,67]. VeroE6 cells were plated at 3000 cells/well in 384 well clear bottom black cell culture plate (Greiner Bio-One). Twenty-four h post seeding, human sera, which were heat-inactivated at 56°C for 30 min, were diluted 1:20 (starting dilution), then serially diluted 2-fold for 8 dilutions in DMEM (2% FBS, 1% sodium pyruvate, and 1% Penicillin-Streptomycin). 50 μL dilutions were added to 50 μL of icSARS-CoV-2-nLuc-GFP (MOI 0.01, ~2PFU/ μL), and incubated for 1 hour at 37°C. Media was then removed from cells and replaced with 20 μL of virus-sera mixture, and incubated for 48 h at 37°C. 5 μL of Nano-Glo Luciferase substrate (Promega) was added to each well and luciferase signal was measured using Cytation 5 plate reader (BioTek). Values of serum-treated samples were normalized to non-serum controls. Assay was performed in triplicate and averages of these normalized values were plotted in Prism 9 (GraphPad). IC_50_ values were calculated.

### Western blotting

Expression of SARS-CoV-2 spike and hACE2 proteins in cells were verified by immunoblotting. In the transient system, cells transfected with plasmids encoding Spike or hACE2 were lysed with RIPA buffer 48 h post transfection. Stable cell lines expressing spike or hACE2 were similarly lysed. Samples were boiled for 10 min in the presence of SDS and DTT and size-separated on a pre-made SDS-PAGE gradient gel (Bio-Rad), which was then transferred onto PVDF filter membranes as described [65]. hACE2 and Spike proteins were detected by goat anti-hACE2 and anti-FLAG primary and rabbit anti-goat-HRP and rabbit anti-mouse-HRP secondary antibodies, respectively.

## Supporting information

S1 Fig Syncytia formation and luciferase activity after transient transfection. 293T cells were transiently co-transfected with increasing amounts of CMV expression plasmids separately encoding S and hACE2, along with CMV-Tat and HIV LTR-FFLUC. At 48 h cells were fixed, stained with crystal violet (A-E), and photomicrographed, with semi-quantification of cell syncytia indicated (- rare or no syncytia; +++ most cells are in syncytia). In parallel RLU was measured, +/- SD (F).
(TIF)

S2 Fig Syncytia formation and luciferase activity after transient transfection. 293T cells were transiently transfected either with CMV expression plasmids encoding S and CMV-Tat or hACE2 and LTR-FFLUC in increasing amounts (0.5–4.0 μg per well). Cells were mixed 48 h post transfection. Plates were microphotographed 24 h post co-incubation after fixation and crystal violet staining (A-E); in parallel RLU +/- SD was measured at 24 h post co-incubation (F). Quantification of syncytia as per S1 Fig.
(TIF)

S3 Fig Cell fusion depends upon both receptor and viral glycoprotein. 293Ts were transfected with CMV-Tat and either mock (-), CMV-hACE2 (A), or CMV-TMPRSS2 (P) [indicated at bottom of each bar to left of forward slash (/)], or HIV LTR-FFLUC and either mock (-), CMV-Spike (S), CMV-TMPRSS2 (P) VSV-G (V) [indicated at bottom of each bar to right of forward slash (/)]. At 48 h cells were mixed 1:1 and 48 h later lysed and RLU measured. Background was ~1000 RLU but increased several orders of magnitude in presence of both Spike and hACE2. Addition of protease had no effect or was inhibitory. Note log scale of ordinate axis.
(TIF)

S4 Fig Detection of S and hACE2 protein by immunoblot. (A) hACE2 immunoblots. Lane 1: 293Ts, 2: 293T-hACE2 cells, 3: HOS-3742, 4: HOS-3734, 5: HOS cells. Below is shown immunoblot for GAPDH as a loading control. (B) Spike immunoblots. Lane 1:293Ts, 2: 293Ts transfected with pcDNA-SARS-CoV-2-S, 3: TZMbl-Spike cells, 4: TZMbl-EV cells. Below is shown immunoblot for GAPDH as a loading control.
(TIF)

S5 Fig Syncytia formation and luciferase activity after co-incubation in the stable cell line system. (A-F) Producer cells stably expressing spike protein (TZMbl-Spike) or control cells with empty vector (TZMbl-EV) were mixed with target cells stably expressing hACE2 (HOS-3734/HOS-3742) or control cells (HOS-EV). After 24 h, cells were photomicrographed and syncytia semi-quantified. In parallel, cells were lysed and RLU +/- SD measured (G). Producer TZMbl-Spike and target HOS-3734 cells were mixed in triplicate in 96-well plates, and RLU measured at different time points after co-culture (H). Semi-quantification of cell syncytia as per S1 Fig.
(TIF)

S6 Fig Syncytia were LacZ+. Producer cells stably expressing spike protein (TZMbl-Spike) or control cells with empty vector (TZMbl-EV) were mixed with target cells stably expressing hACE2 (HOS-3734/HOS-3742) or control cells (HOS-EV). After 24 h, cells were fixed in formaldehyde-glutaraldehyde, stained using X-gal at 37°C overnight, and photomicrographed. All syncytia were blue. Semi-quantification of cell syncytia as per S1 Fig.
(TIF)

S7 Fig Time-of-addition experiment using LCB1 to inhibit cell fusion. Four-fold serially diluted LCB1 was added 1 h before (A) or at 0 (B), 1 (C), 2 (D), or 4 h (E) after co-culture of HOS-3734 and TZMbl-S cells. RLU was measured the next day.
(TIF)

S8 Fig COVID-19+ convalescent and acute phase sera inhibit cell fusion in the stable system. (A-F) Four-fold serially-diluted sera were pre-incubated with TZMbl-Spike producer cells for 1 h, then HOS-3734 or HOS-3742 target cells were added. RLU was measured the next day. The red and blue curves represent fitting to data obtained from HOS-3734 and HOS-3742, respectively.
(TIF)

S9 Fig Neutralization of replication-competent Sars-Cov-2 virus with nano luciferase reporter. Fifty μL of indicated two-fold serially diluted sera was mixed with 50 μL of icSars-Cov-2-nLuc-GFP virus (MOI 0.01, ~2 PFU/μL) 1 h before incubation with VeroE6 cells. Nano-Glo Luciferase activity was quantified at 48 h and normalized to no serum control.
(TIF)

S10 Fig Pairwise correlations of IC-50s between cell fusion, pseudotyping, and replication-competent virus assays. (A) Correlation between pseudotyping and cell fusion assays (R^2^ = 0.9968). (B) Correlation between pseudotyping and replication-competent virus assays (R^2^ = 0.8206). (C) Correlation between cell fusion and replication-competent virus assays (R^2^ = 0.788). In all cases plotted values reflect serum titer required to achieve 50% inhibition of RLU activity.
(TIF)

S11 Fig Reproducibility of cell fusion assay. (A-E) Inhibition of cell fusion by LCB1 was performed in 5 independent experiments and in each case IC_50_ values were calculated. (F) Overall reproducibility of IC-50 values of the cell fusion assay using LCB1 peptide.
(TIF)

S1 Table Demographic and clinical characteristics of the COVID-19+ subjects. (DOCX)
