# Supplementary figures and images for: Rapid, reliable, and reproducible cell fusion assay to quantify SARS-Cov-2 spike interaction with hACE2
Source: PLoS Pathog. 2021 Jun 24;17(6):e1009683. doi: 10.1371/journal.ppat.1009683 (PMC8263067; doi:10.1371/journal.ppat.1009683)

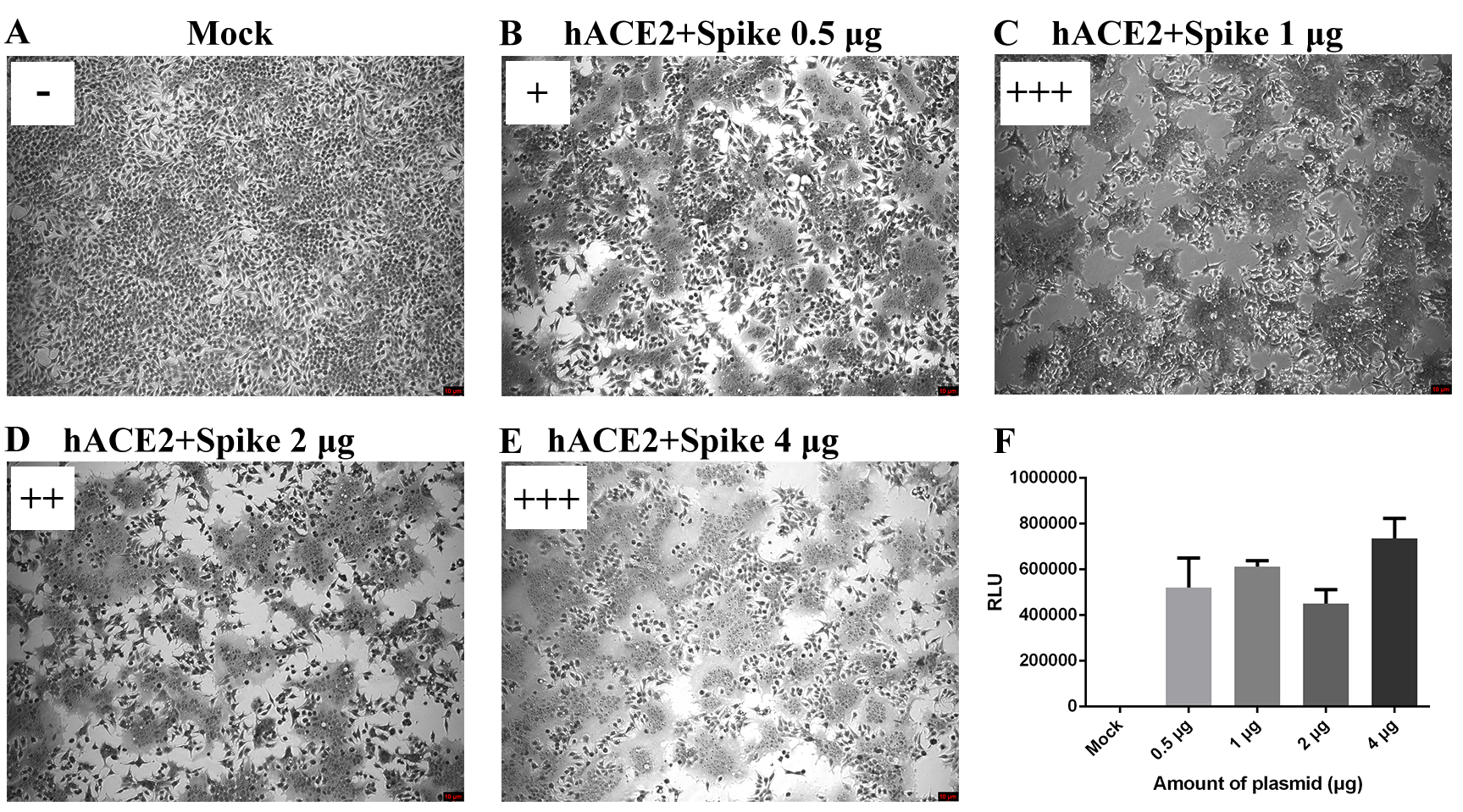

Supplement: S1 Fig — 293T cells were transiently co-transfected with increasing amounts of CMV expression plasmids separately encoding S and hACE2, along with CMV-Tat and HIV LTR-FFLUC. At 48 h cells were fixed, stained with crystal violet (A-E), and photomicrographed, with semi-quantification of cell syncytia indicated (- rare or no syncytia; +++ most cells are in syncytia). In parallel RLU was measured, +/- SD (F). (TIF) [file ppat.1009683.s001.tif]

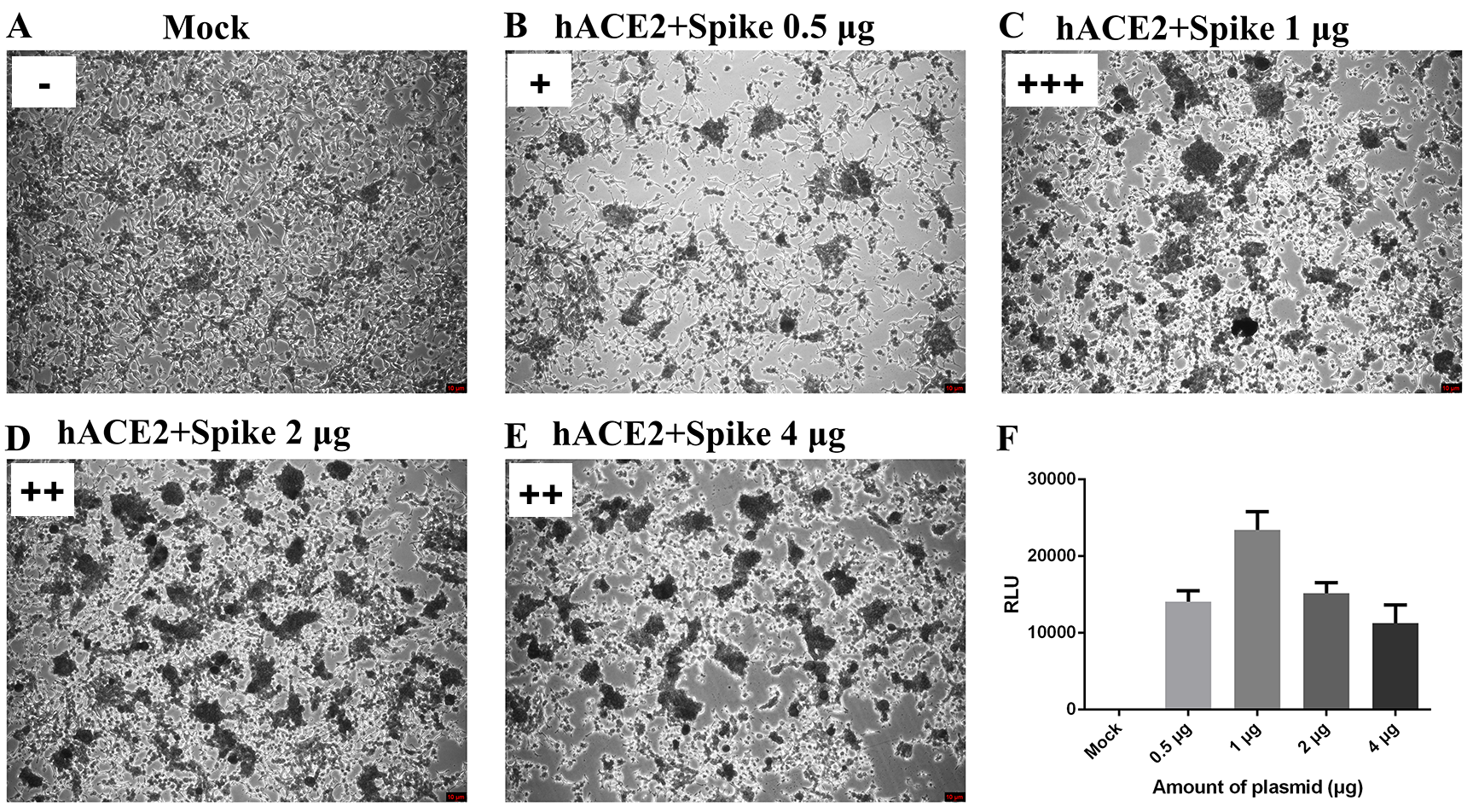

Supplement: S2 Fig — 293T cells were transiently transfected either with CMV expression plasmids encoding S and CMV-Tat or hACE2 and LTR-FFLUC in increasing amounts (0.5–4.0 μg per well). Cells were mixed 48 h post transfection. Plates were microphotographed 24 h post co-incubation after fixation and crystal violet staining (A-E); in parallel RLU +/- SD was measured at 24 h post co-incubation (F). Quantification of syncytia as per S1 Fig. (TIF) [file ppat.1009683.s002.tif]

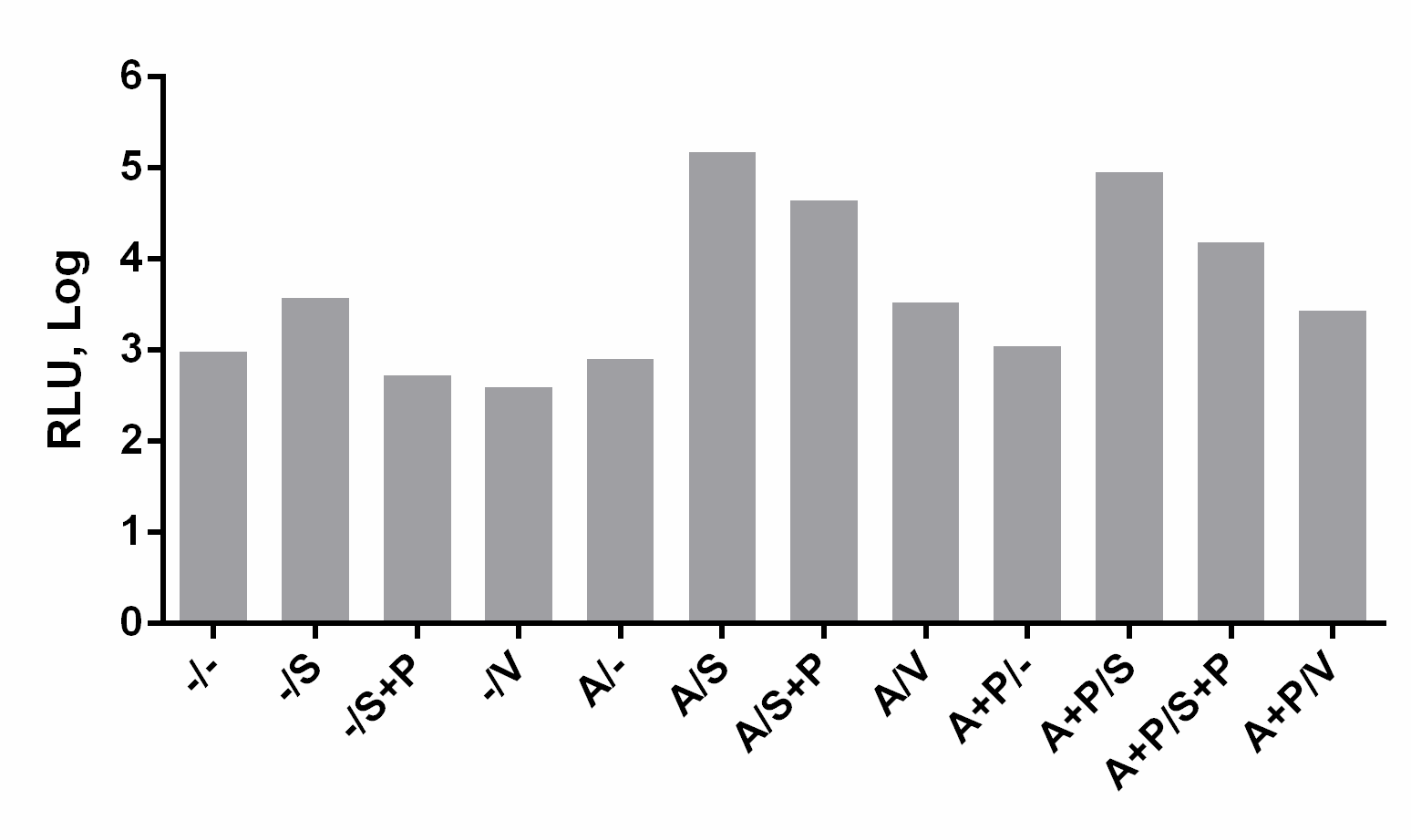

Supplement: S3 Fig — 293Ts were transfected with CMV-Tat and either mock (-), CMV-hACE2 (A), or CMV-TMPRSS2 (P) [indicated at bottom of each bar to left of forward slash (/)], or HIV LTR-FFLUC and either mock (-), CMV-Spike (S), CMV-TMPRSS2 (P) VSV-G (V) [indicated at bottom of each bar to right of forward slash (/)]. At 48 h cells were mixed 1:1 and 48 h later lysed and RLU measured. Background was ~1000 RLU but increased several orders of magnitude in presence of both Spike and hACE2. Addition of protease had no effect or was inhibitory. Note log scale of ordinate axis. (TIF) [file ppat.1009683.s003.tif]

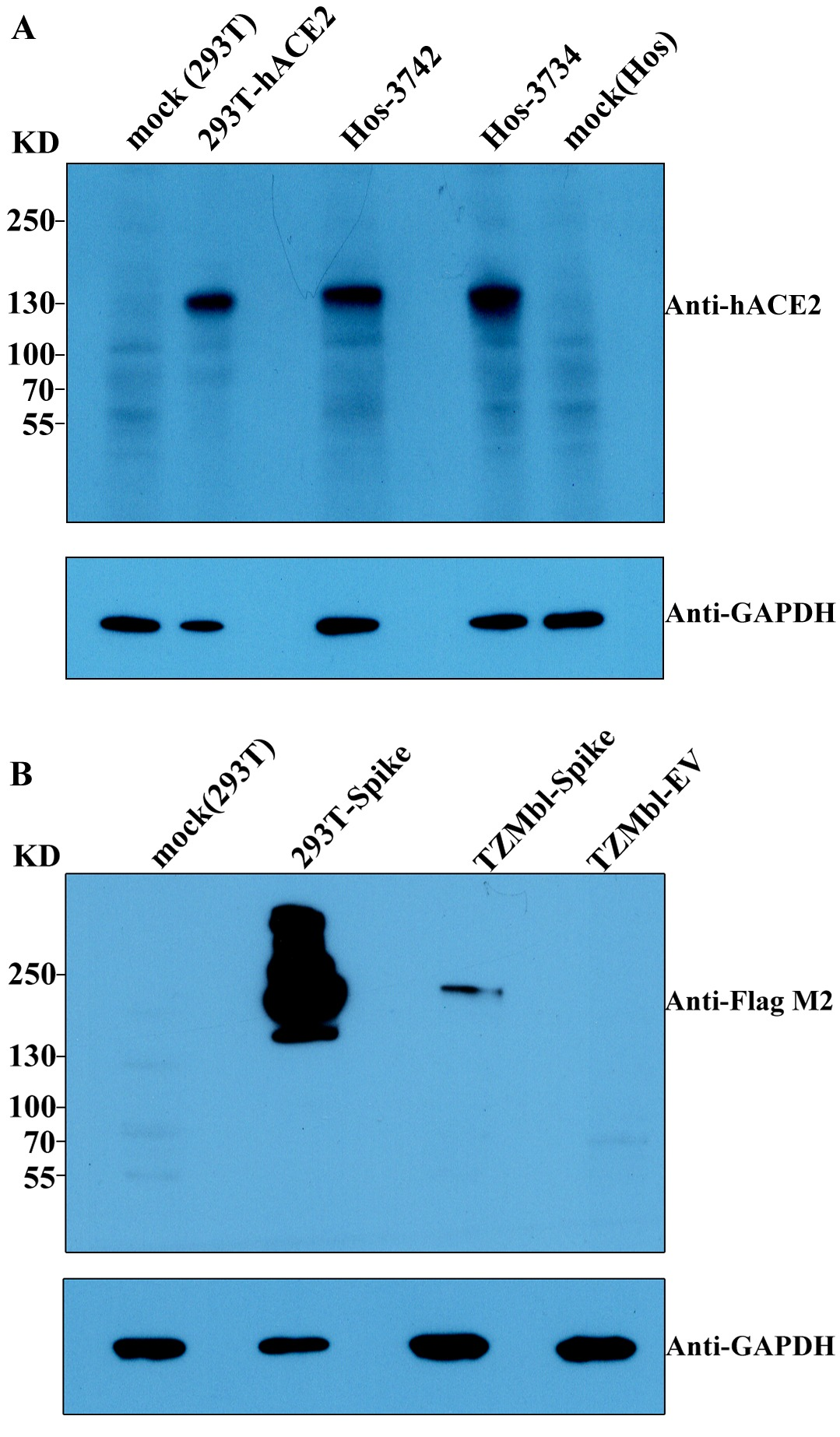

Supplement: S4 Fig — (A) hACE2 immunoblots. Lane 1: 293Ts, 2: 293T-hACE2 cells, 3: HOS-3742, 4: HOS-3734, 5: HOS cells. Below is shown immunoblot for GAPDH as a loading control. (B) Spike immunoblots. Lane 1:293Ts, 2: 293Ts transfected with pcDNA-SARS-CoV-2-S, 3: TZMbl-Spike cells, 4: TZMbl-EV cells. Below is shown immunoblot for GAPDH as a loading control. (TIF) [file ppat.1009683.s004.tif]

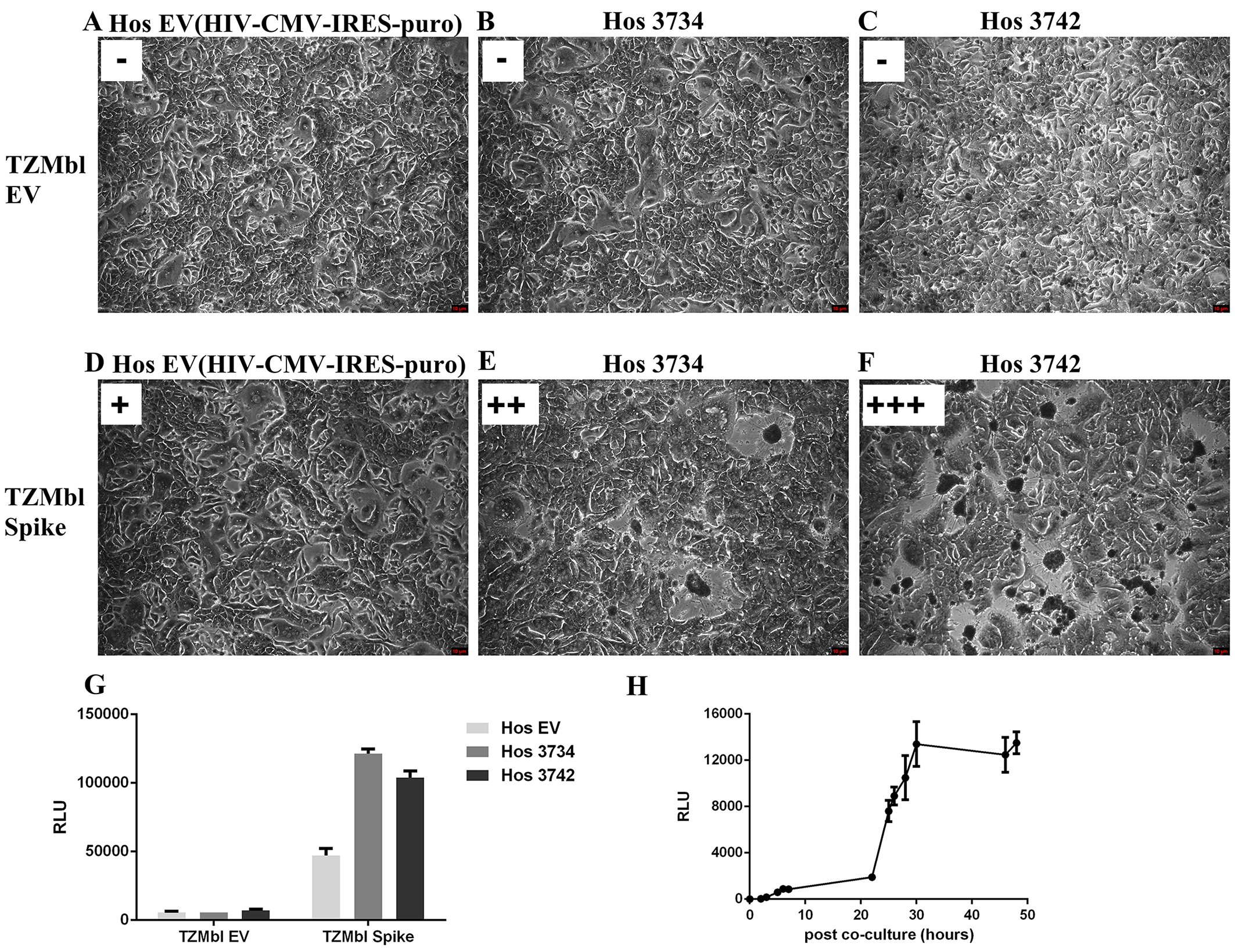

Supplement: S5 Fig — (A-F) Producer cells stably expressing spike protein (TZMbl-Spike) or control cells with empty vector (TZMbl-EV) were mixed with target cells stably expressing hACE2 (HOS-3734/HOS-3742) or control cells (HOS-EV). After 24 h, cells were photomicrographed and syncytia semi-quantified. In parallel, cells were lysed and RLU +/- SD measured (G). Producer TZMbl-Spike and target HOS-3734 cells were mixed in triplicate in 96-well plates, and RLU measured at different time points after co-culture (H). Semi-quantification of cell syncytia as per S1 Fig. (TIF) [file ppat.1009683.s005.tif]

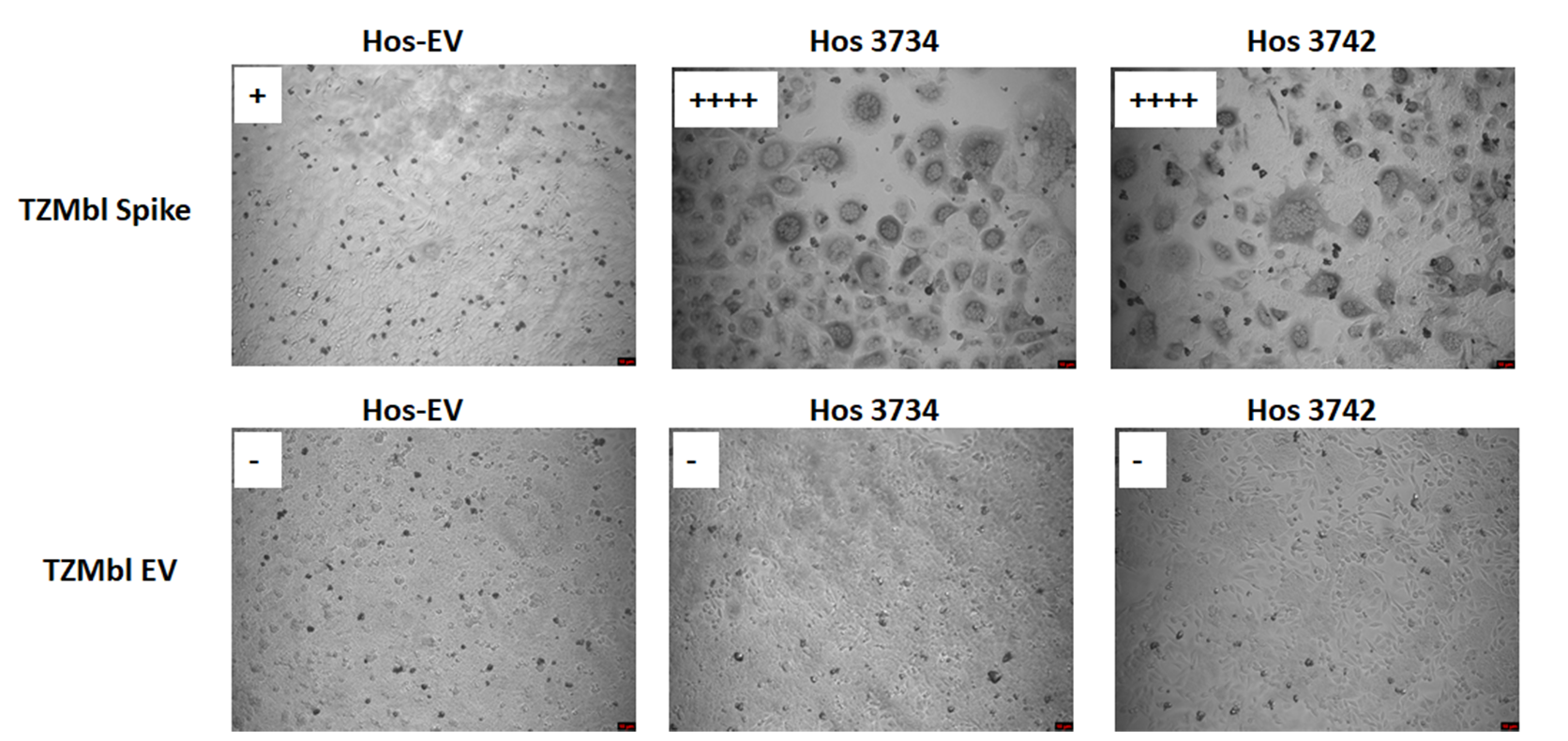

Supplement: S6 Fig — Producer cells stably expressing spike protein (TZMbl-Spike) or control cells with empty vector (TZMbl-EV) were mixed with target cells stably expressing hACE2 (HOS-3734/HOS-3742) or control cells (HOS-EV). After 24 h, cells were fixed in formaldehyde-glutaraldehyde, stained using X-gal at 37°C overnight, and photomicrographed. All syncytia were blue. Semi-quantification of cell syncytia as per S1 Fig. (TIF) [file ppat.1009683.s006.tif]

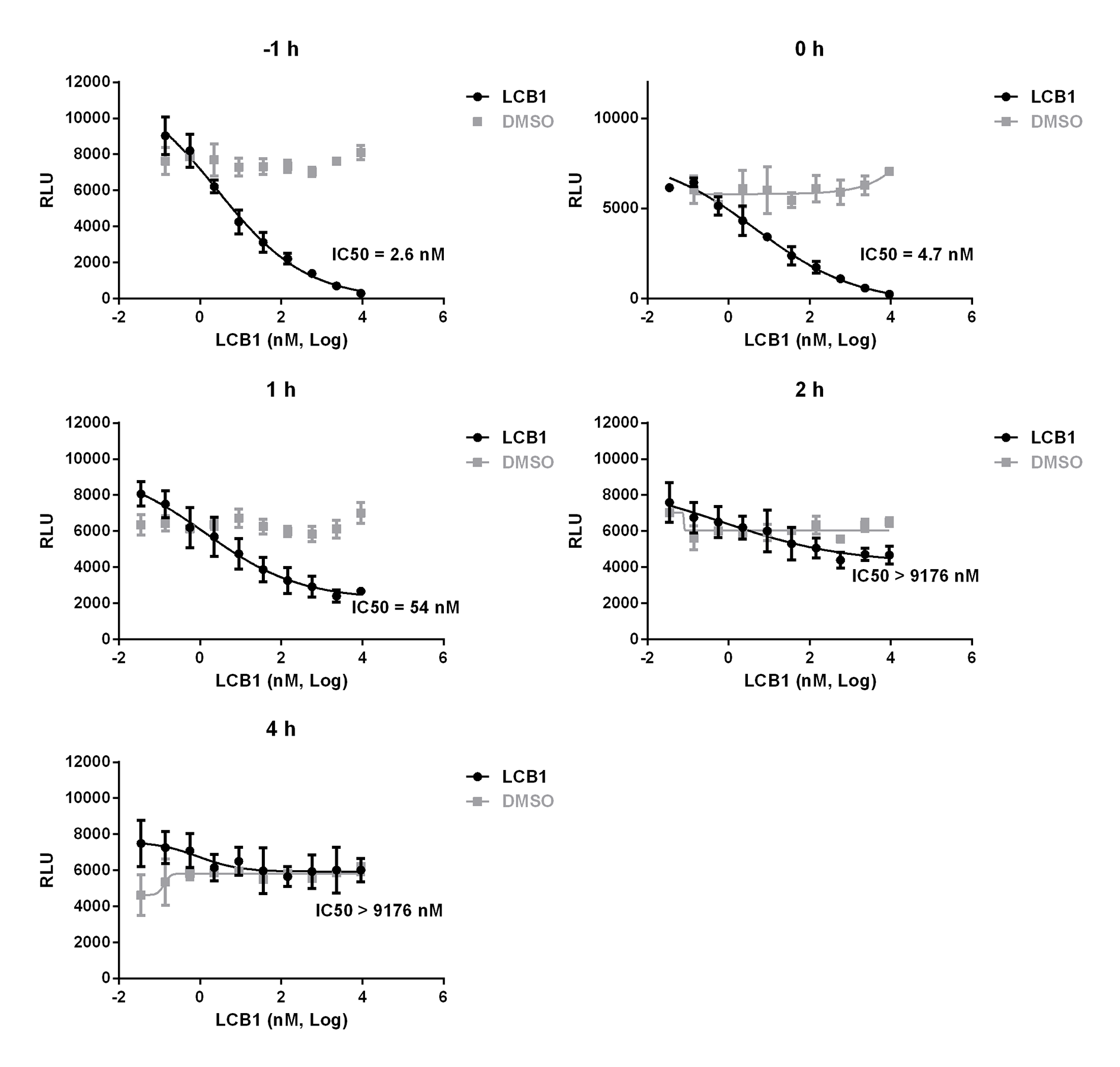

Supplement: S7 Fig — Four-fold serially diluted LCB1 was added 1 h before (A) or at 0 (B), 1 (C), 2 (D), or 4 h (E) after co-culture of HOS-3734 and TZMbl-S cells. RLU was measured the next day. (TIF) [file ppat.1009683.s007.tif]

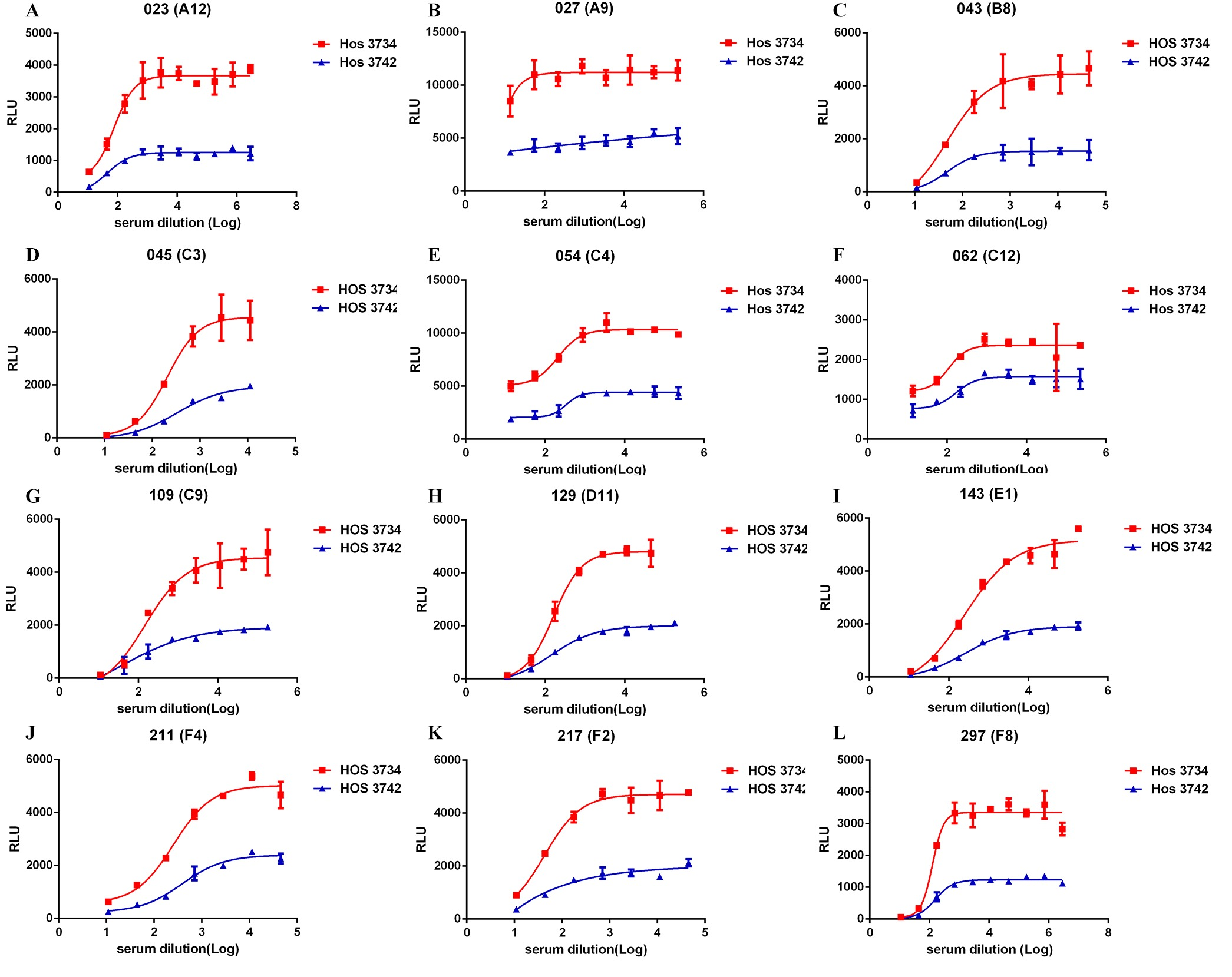

Supplement: S8 Fig — (A-F) Four-fold serially-diluted sera were pre-incubated with TZMbl-Spike producer cells for 1 h, then HOS-3734 or HOS-3742 target cells were added. RLU was measured the next day. The red and blue curves represent fitting to data obtained from HOS-3734 and HOS-3742, respectively. (TIF) [file ppat.1009683.s008.tif]

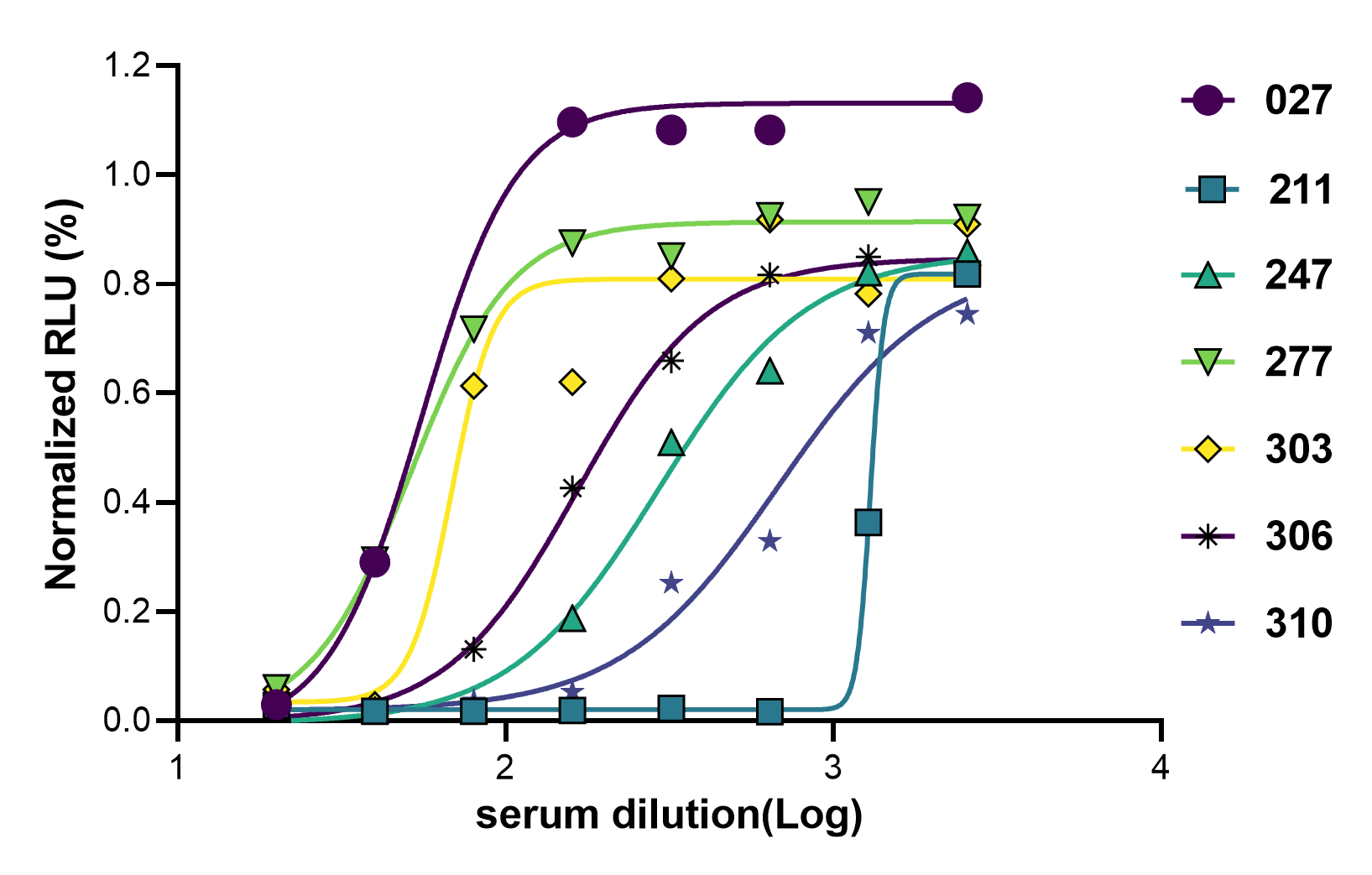

Supplement: S9 Fig — Fifty μL of indicated two-fold serially diluted sera was mixed with 50 μL of icSars-Cov-2-nLuc-GFP virus (MOI 0.01, ~2 PFU/μL) 1 h before incubation with VeroE6 cells. Nano-Glo Luciferase activity was quantified at 48 h and normalized to no serum control. (TIF) [file ppat.1009683.s009.tif]

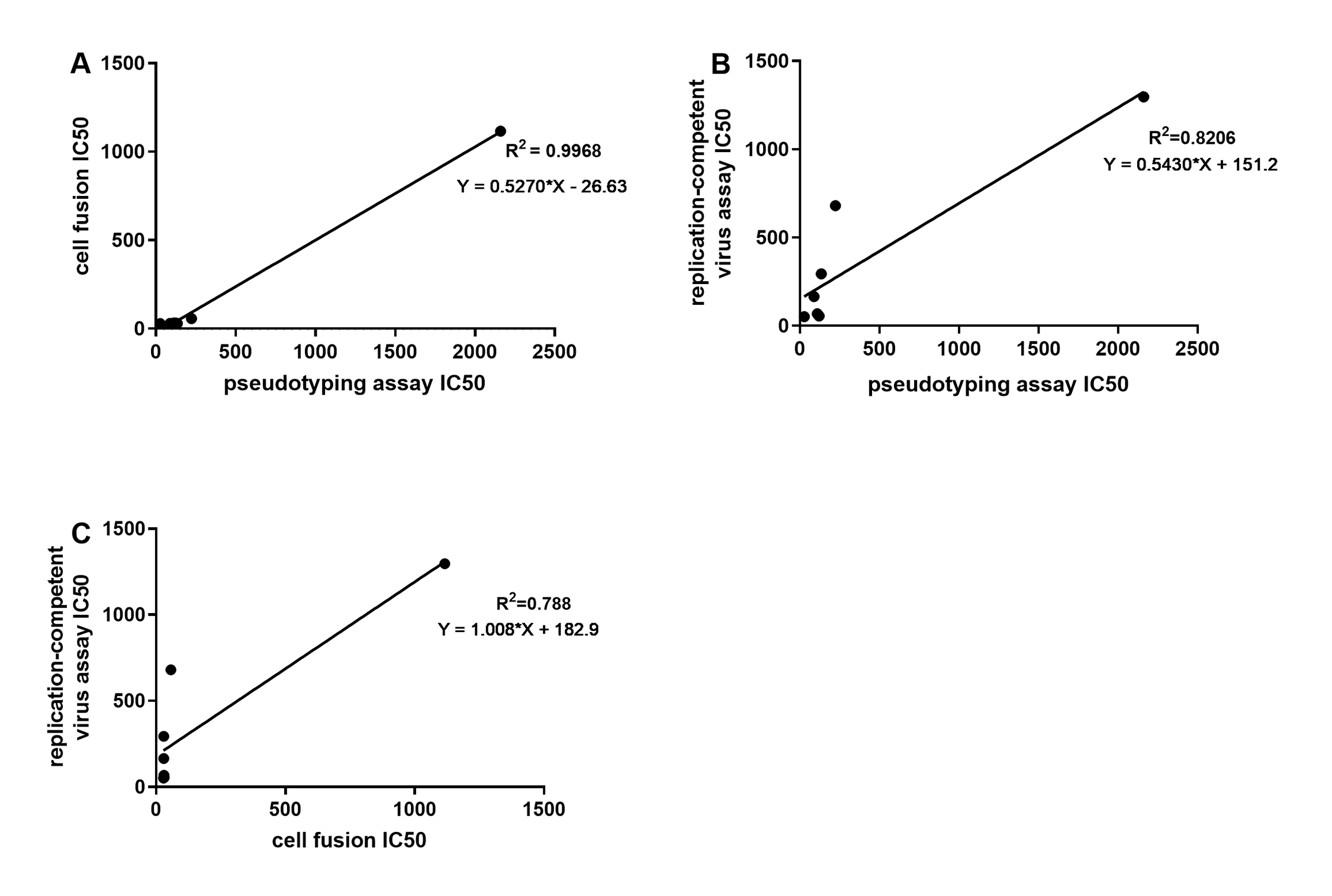

Supplement: S10 Fig — (A) Correlation between pseudotyping and cell fusion assays (R2 = 0.9968). (B) Correlation between pseudotyping and replication-competent virus assays (R2 = 0.8206). (C) Correlation between cell fusion and replication-competent virus assays (R2 = 0.788). In all cases plotted values reflect serum titer required to achieve 50% inhibition of RLU activity. (TIF) [file ppat.1009683.s010.tif]

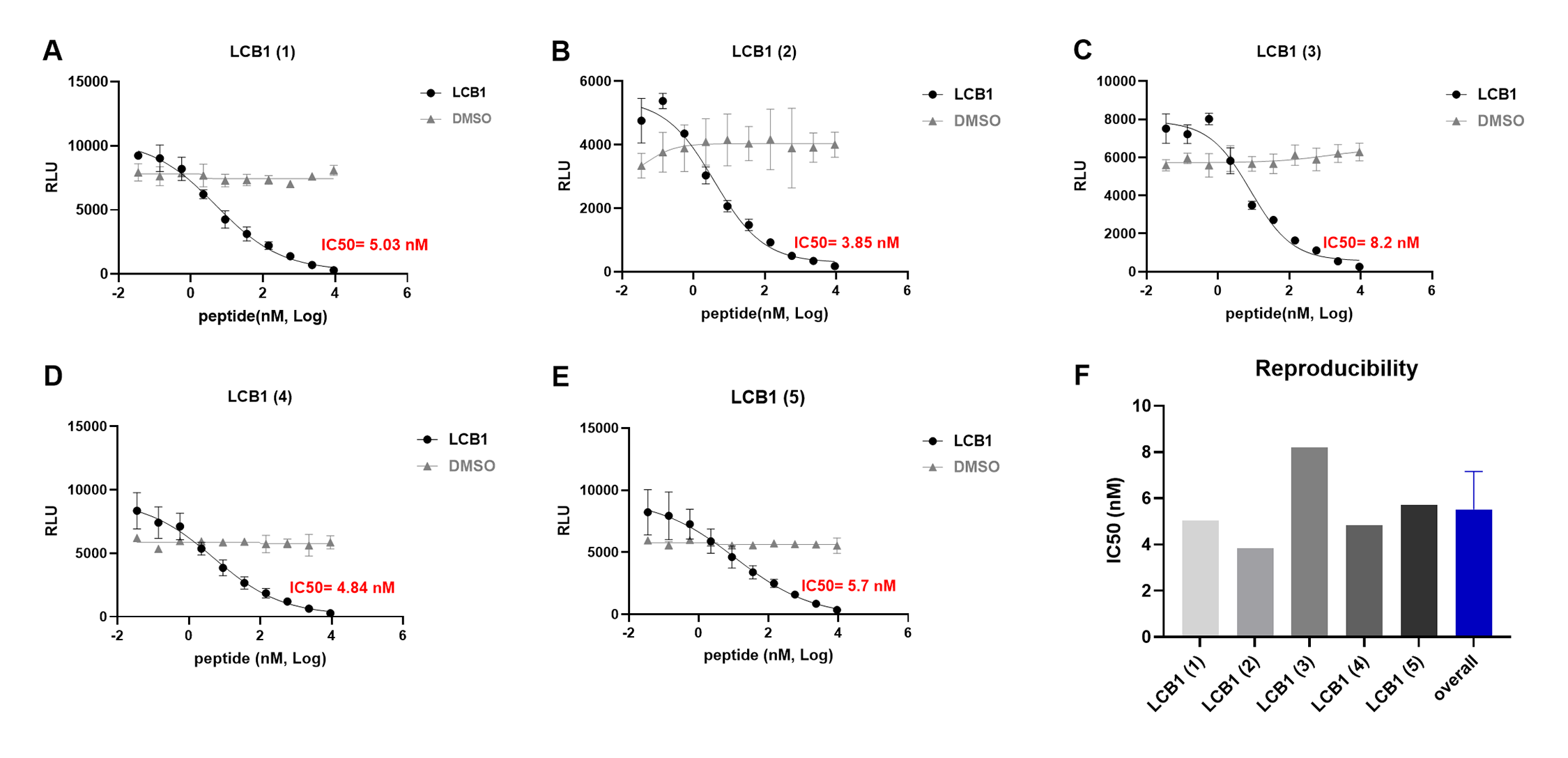

Supplement: S11 Fig — (A-E) Inhibition of cell fusion by LCB1 was performed in 5 independent experiments and in each case IC50 values were calculated. (F) Overall reproducibility of IC-50 values of the cell fusion assay using LCB1 peptide. (TIF) [file ppat.1009683.s011.tif]
