# Supplementary material for: Rapid, reliable, and reproducible cell fusion assay to quantify SARS-Cov-2 spike interaction with hACE2
Source: PLoS Pathog. 2021 Jun 24;17(6):e1009683. doi: 10.1371/journal.ppat.1009683 (PMC8263067; doi:10.1371/journal.ppat.1009683)
Supplement: S1 Table — (DOCX) [file ppat.1009683.s012.docx]

Demographic and clinical characteristics of the COVID-19+ subjects

| Subject ID | Date of sample collection (after symptom onset) | Age | Sex | Race | BMI^1^ | Co-morbidities | | | | Severity of disease | | Outcome |
| --- | --- | --- | --- | --- | --- | --- | --- | --- | --- | --- | --- | --- |
|  |  |  |  |  |  | Chronic heart disease? | Chronic lung disease? | High blood pressure | Other co-morbid conditions | moderate | severe |  |
| 0023 | 17 | 42 | M | white | 44 |  |  |  |  |  | intubated^4^ | D/C^5^ post 1 month |
| 0027 | 13 | 70 | M | white | 28.8 |  |  | + |  | floor^3^ |  | D/C post 3 weeks |
| 0043 | 12 | 65 | F | black | 43.9 |  |  | + | diabetes |  | intubated | D/C post 2 months |
| 0045 | 21 | 62 | F | black | 34.2 | CHF^2^ | asthma | + | diabetes |  | intubated | D/C post 3 months |
| 0054 | 18 | 72 | M | hispanic | 34.4 |  |  | + | prostate cancer |  | intubated | deceased post 1 month |
| 0062 | 28 | 32 | M | hispanic | 31 |  |  | + |  |  | intubated, ECMO | D/C post 2.5 months |
| 0109 | 18 | 84 | F | hispanic | 46 | CHF | asthma | + | diabetes, CKD^6^ | floor |  | D/C post 2 weeks |
| 0129 | 52 | 67 | M | white | 33.8 |  |  | + |  |  | intubated | D/C post 1 month |
| 0143 | 24 | 77 | F | white | 22.9 | Afib^11^ | asthma | + | breast and esophageal cancer | floor |  | D/C post 1 month |
| 0211 | 46 | 62 | F | white | 39.8 | CAD^12^ |  | + | diabetes, ESRD^7^ |  | BIPAP^10^ | D/C post 5 weeks |
| 0217 | 21 | 76 | F | white | 25.3 |  | COPD^13^ | + | UC^8^ | floor |  | D/C post 1 month |
| 0297 | 40 | 70 | M | white | 48.8 | CHF, Afib | COPD | + | OSA^9^ |  | intubated | D/C post 2 months |
| 0247 | 8 | 80 | F | black | 47.9 |  |  | + | diabetes |  | intubated | D/C post 2 weeks |
| 0277 | 7 | 40 | M | black | 62 |  |  |  |  | floor |  | D/C post 1 week |
| 0303 | 15 | 78 | F | white | 31.8 | Afib, Aortic Stenosis |  | + | Rheumatoid Arthritis | floor |  | D/C post 1 week |
| 0306 | 8 | 66 | M | black | 33.4 |  |  |  | diabetes |  | intubated | D/C post 1 month |
| 0310 | 13 | 45 | F | Other, non hispanic | 29.05 |  |  |  | Familial hypercholesterolemia |  | BIPAP | D/C post 1.5 weeks |
| Notes:  ^1^BMI denotes body mass index  ^2^CHF denotes congestive heart failure  ^3^Floor denotes subject remained on a regular in-patient unit  ^4^Intubated denotes subject was endotracheally intubated in the intensive care unit  ^5^D/C denotes discharge from the hospital  ^6^CKD denotes chronic kidney disease  ^7^ESRD denotes end-stage renal disease requiring dialysis  ^8^UC denotes ulcerative colitis  ^9^OSA denotes obstructive sleep apnea  ^10^BIPAP denotes subject required Bilevel Positive Airway Pressure but not intubation  ^11^Afib denotes atrial fibrillation  ^12^CAD denotes coronary artery disease  ^13^COPD denotes chronic obstructive pulmonary disease | | | | | | | | | | | | |
